# Analysis of the Geometric and Electronic Structure of Spin-Coupled Iron–Sulfur Dimers with Broken-Symmetry DFT: Implications for FeMoco

**DOI:** 10.1021/acs.jctc.1c00753

**Published:** 2022-02-15

**Authors:** Bardi Benediktsson, Ragnar Bjornsson

**Affiliations:** † Science Institute, University of Iceland, Dunhagi 3, 107 Reykjavik, Iceland; ‡ Max-Planck Institute for Chemical Energy Conversion, Stiftstrasse 34-36, 45470 Mülheim an der Ruhr, Germany

## Abstract

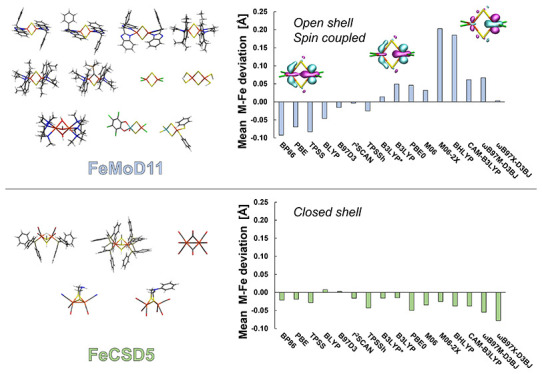

The open-shell electronic structure of iron–sulfur clusters presents considerable challenges to quantum chemistry, with the complex iron–molybdenum cofactor (FeMoco) of nitrogenase representing perhaps the ultimate challenge for either wavefunction or density functional theory. While broken-symmetry density functional theory has seen some success in describing the electronic structure of such cofactors, there is a large exchange–correlation functional dependence in calculations that is not fully understood. In this work, we present a geometric benchmarking test set, FeMoD11, of synthetic spin-coupled Fe–Fe and Mo–Fe dimers, with relevance to the molecular and electronic structure of the Mo-nitrogenase FeMo cofactor. The reference data consists of high-resolution crystal structures of metal dimer compounds in different oxidation states. Multiple density functionals are tested on their ability to reproduce the local geometry, specifically the Fe–Fe/Mo–Fe distance, for both antiferromagnetically coupled and ferromagnetically coupled dimers via the broken-symmetry approach. The metal–metal distance is revealed not only to be highly sensitive to the amount of exact exchange in the functional but also to the specific exchange and correlation functionals. For the antiferromagnetically coupled dimers, the calculated metal–metal distance correlates well with the covalency of the bridging metal–ligand bonds, as revealed via the corresponding orbital analysis, Hirshfeld S/Fe charges, and Fe–S Mayer bond order. Superexchange via bridging ligands is expected to be the dominant interaction in these dimers, and our results suggest that functionals that predict accurate Fe–Fe and Mo–Fe distances describe the overall metal–ligand covalency more accurately and in turn the superexchange of these systems. The best performing density functionals of the 16 tested for the FeMoD11 test set are revealed to be either the nonhybrid functionals r^2^SCAN and B97-D3 or hybrid functionals with 10–15% exact exchange: TPSSh and B3LYP*. These same four functionals are furthermore found to reproduce the high-resolution X-ray structure of FeMoco well according to quantum mechanics/molecular mechanics (QM/MM) calculations. Almost all nonhybrid functionals systematically underestimate Fe–Fe and Mo–Fe distances (with r^2^SCAN and B97-D3 being the sole exceptions), while hybrid functionals with >15% exact exchange (including range-separated hybrid functionals) overestimate them. The results overall suggest r^2^SCAN, B97-D3, TPSSh, and B3LYP* as accurate density functionals for describing the electronic structure of iron–sulfur clusters in general, including the complex FeMoco cluster of nitrogenase.

## Introduction

Nature utilizes complex polynuclear spin-coupled cofactors to carry out complex chemical transformations with the reduction of dinitrogen to ammonia being a prime example. The iron–molybdenum cofactor of the Mo nitrogenase enzyme (FeMoco) features 8 metal ions in Fe(II) and Fe(III) oxidation states, 41 unpaired electrons, spin-polarized covalent Fe–S, Mo–S, and Fe–C metal–ligand bonds; unusual ligand environments (e.g., interstitial carbide); and an unusual spin-coupled Mo(III).^1^ The cofactor has been extensively characterized by X-ray crystallography,^2^ electron paramagnetic resonance (EPR),^3−6^^57^Fe Mössbauer,^7^ X-ray absorption, and X-ray emission spectroscopy,^8−15^ yet details still remain to be uncovered about the nature of the electronic structure such as the local Fe oxidation states, spin coupling, and spin delocalization.^6^ These complex electronic structure properties are likely behind the unique reactivity of the cluster. While theory has played an important role in unraveling the molecular and electronic structure of FeMoco^9,11,16−23^ and similar iron–sulfur clusters^24−27^ (and multiple density functional theory (DFT) studies have suggested possible reaction mechanisms of dinitrogen reduction),^28−35^ much uncertainty remains about how well theory describes the complicated electronic structure that these clusters exhibit.

The simplest spin-coupled systems already pose a challenge to contemporary quantum chemistry. An antiferromagnetically coupled singlet state cannot be fully described by a single-determinant wavefunction. Instead, one must settle for a symmetry-broken spin-contaminated *M*_S_ = 0 unrestricted Hartree–Fock (HF) state that features unphysical localized spin density present on each spin center (α and β spin density, respectively), while the exact *S* = 0 state has zero spin density everywhere in space. This lack of a spin eigenfunction in the reference is an inconvenient starting point for a post-HF approach, and this problem is arguably only satisfactorily dealt with at the multireference wavefunction level where a spin-adapted multiconfigurational reference can be used instead. Alternatively, spin projection of spin-symmetry-broken states via the use of model Hamiltonians (e.g., Heisenberg–Dirac–Van Vleck, HDVV) can be used to correct the energy of the low-spin state, and this strategy has recently been used to correct coupled-cluster calculations utilizing a broken-symmetry UHF reference.^36^

Spin-adapted multireference calculations should allow the most satisfactory treatment of spin-coupled systems. However, there are challenges associated with treating a large enough active space in the complete active space self-consistent field (CASSCF) reference calculation, and even more difficult challenges in the subsequent dynamic correlation treatment. Large active space CASSCF calculations that use approximations to the full configuration interaction (FCI) problem within the active space are beginning to emerge for iron–sulfur systems.^27,37−42^ Examples include: calculations based on the density matrix renormalization group, DMRG-CASSCF,^27^ and FCI quantum Monte Carlo^42^ that have been applied to the simplest [2Fe–2S] dimers as well as [4Fe–4S] clusters. Recently, DMRG-CASSCF calculations of the large Fe_8_S_7_ cluster of the MoFe protein (P-cluster) were performed with active spaces of up to 120 electrons in 77 orbitals, shedding light on the complex dense low-energy spectrum of this complex cluster.^41^ CASSCF calculations of FeMoco with active spaces up to 113 electrons in 76 orbitals have recently been achieved.^38^ These studies have revealed that spin-coupled iron–sulfur systems feature a large number of low-energy electronic states, more than assumed in effective spin Hamiltonians (HDVV as well as the extended double-exchange version HDE).^27^ While it is encouraging that large active space CASSCF calculations are becoming possible for systems as large as the P-cluster and FeMoco, questions remain about the accuracy of these results as dynamic correlation effects are typically unaccounted for in these calculations,^43^ yet they would be important for capturing the covalency of the iron–sulfur chemical bonds. Unfortunately, there are theoretical problems with applying multireference perturbation theory to large active space CASSCF references,^44,45^ and more robust multireference configuration interaction (MRCI) or coupled-cluster (MRCC) approaches typically remain out of reach.

An alternative to the multireference wavefunction approach comes from unrestricted density functional theory. Kohn–Sham density functional theory (KS-DFT) bypasses the calculation of the wavefunction of the system and assumes instead that a single-determinant description of a noninteracting reference system together with an exchange–correlation energy functional is sufficient to describe the electron density and energy of any system of interest. The single-determinant nature of KS-DFT implies at first glance that it should suffer from the same problem as a single-determinant HF wavefunction with unphysical spin density for an *S* = 0 system.^46^ However, the extent of this problem remains unclear since the total spin operator operates on the noninteracting KS reference system instead of the full interacting system, with the spin of the system thus not well defined. KS-DFT is typically considered an exact approach (although this rests on the assumption that the density is always noninteracting *v*-representable),^47−53^ which implies that an exact Kohn–Sham density functional calculation should give the exact energy of a system, even though the noninteracting reference system clearly breaks spin symmetry.^54^ Approximate spin projection schemes, e.g., based on the Yamaguchi^55,56^ or the Noodleman^57−59^ equations, are commonly applied to correct for the spin contamination of the low-spin state. This is performed using the energies of the antiferromagnetic broken-symmetry solution and the ferromagnetic solution to parameterize an effective Hamiltonian such as the HDVV. This allows one to derive the energy of the true uncontaminated *S* = 0 spin state. Such spin projection schemes have been reasonably successful in many studies,^60^ leading to qualitatively and often semiquantitatively correct results, albeit with a large functional dependence.^61^

The ambiguous nature of the spin-contaminated broken-symmetry state poses a theoretical problem for structural optimizations of spin-coupled systems, with some practitioners preferring to optimize the structure of the less spin-contaminated ferromagnetic state rather than the broken-symmetry state. This approach seems justified in cases of weak spin-coupling where geometries of ferromagnetic and antiferromagnetic states have been found to be very similar, e.g., for Mn–O dimers.^62^ This is not the case for Fe–S systems (see, e.g., refs (37) and (63)) and is discussed later. A pragmatic alternative is to instead optimize the geometries of spin-coupled systems using the broken-symmetry determinant and assume thereby that the broken-symmetry state is an accurate enough representation of the spin-coupled low-spin state and that all important correlation effects are included via the exchange–correlation functional. This approach has been utilized by us and others in various DFT and DFT/molecular mechanics (MM) studies on the multimetal spin-coupled FeMoco and FeVco (iron–vanadium cofactor of vanadium nitrogenase) clusters where excellent agreement with the high-resolution crystal structure has been obtained.^22,64−66^ In fact, the strong correlation between the experimental Fe–Fe and Mo–Fe distances of FeMoco and BS-DFT-calculated Fe–Fe and Mo–Fe distances implies that the BS-DFT states calculated might be considered quite reasonable approximations to the true electronic states. As recently discussed in the literature, however, there is a large functional dependence in BS-DFT calculations on FeMoco, and the functional choice strongly affects both the structure of the cofactor and reaction energies.^28,29,67,68^ In previous work,^29^ we have argued that hybrid functionals with >20% exact exchange lead to unacceptable structural deviations (systematic overestimations) for FeMoco compared to the high-resolution (1.0 Å) crystal structure.^2^ In the case of nonhybrid functionals, these functionals systematically underestimate the Fe–Fe and Mo–Fe distances instead. TPSSh, a 10% exact exchange hybrid functional, was found to give the most satisfactory description of the molecular structure of FeMoco of tested functionals.^29^

Finally, we note a recent alternative approach: approximate spin projection correction of gradients via the extended broken-symmetry (EBS) method.^69^ The extended broken-symmetry approach employs approximate spin projection to the nuclear gradient, and the method has been applied to structural optimizations and even vibrational frequencies of iron–sulfur systems.^69,63,71,70^ This approach assumes the validity of a specific model Hamiltonian (e.g., HDVV), which may present problems if the model Hamiltonian does not give a realistic description of the system, a problem that has been discussed for iron–sulfur systems.^27^

Systematic structural benchmarking studies of metal complexes, such as those by Bühl and co-workers^72−74^ using gas-phase electron diffraction and microwave spectroscopy reference data, have been popular in the literature and continue to be used to test different density functional approximations. Importantly, these test sets feature exclusively metal complexes with closed-shell electronic structure and fewer studies include test sets featuring complexes with open-shell electronic structure. Szilagyi and Winslow investigated spin-coupled iron–sulfur complexes and showed that the geometry of a [Fe_2_S_2_(SPh)_4_]^2–^ dimer was sensitive to both basis set and functional choice. Their results indicated that a 5% hybrid functional (B5HFP) gave accurate spin density distributions,^75^ and in a later study, Harris and Szilagyi demonstrated that a 5% hybrid functional also gave reasonable Fe–S covalency.^76^ Moreover, Noodleman and co-workers demonstrated that accurate geometries of spin-coupled iron–sulfur systems are essential for accurate ^57^Fe Mössbauer parameters.^77^ Guidoni and co-workers, testing both BS-DFT and EBS-DFT geometry optimizations found, on the other hand, that B3LYP yielded reasonable vibrational frequencies, but geometries optimized with the M06 functional gave structures in best agreement with experiment.^63,70^

**Figure 1:**
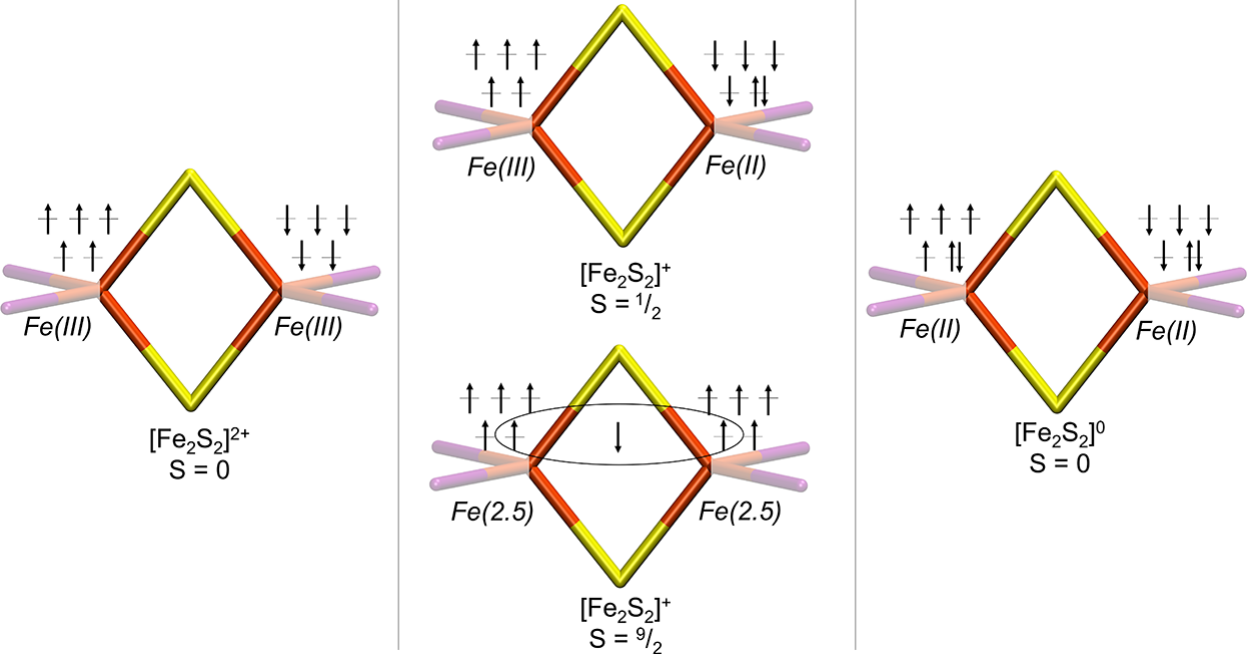
Spin-coupled redox states exhibited by the [2Fe–2S] core as representative for the FeMoD11 test set. Orange indicates iron, yellow indicates sulfur, and purple is a terminal ligand.

In this work, we study the molecular and electronic structure of spin-coupled Fe–S systems (see Figure 1) as described by broken-symmetry DFT structural optimizations, focusing especially on the functional dependence and how the electronic structure of these systems influences the molecular structure. We introduce a test set of 11 complexes, FeMoD11, which includes eight antiferromagnetically spin-coupled Fe–Fe dimers, one ferromagnetically spin-coupled Fe–Fe dimer, and two antiferromagnetically coupled Mo–Fe dimers, inspired by dimeric fragments found in FeMoco. The test set features antiferromagnetic interactions (via bridging ligand superexchange), as well as double-exchange interactions (via direct d-overlap), both known to be important features in the electronic structure of iron–sulfur clusters such as FeMoco. We show that the spin-coupled systems have completely different functional dependencies compared to closed-shell systems and discuss how the metal–metal distance depends strongly on the covalency of bridging metal–ligand bonds in spin-coupled metal dimers. The implications for the BS-DFT description of FeMoco are discussed, and we extend the functional comparison to a quantum mechanics/molecular mechanics (QM/MM) model of FeMoco.

## Computational Details

All X-ray crystal structures were downloaded from the Cambridge Crystallographic Data Centre^78^ and were used as the starting structure for geometrical optimizations. Where missing, hydrogens were added manually. All calculations were performed with the ORCA quantum chemistry program package version 4.2.1^79^ (unless otherwise stated). The self-consistent field (SCF) convergence criteria were set to 10^–8^ Eh (energy change), and tight optimization criteria were used (energy change of 10^–6^ Eh, root-mean-square (RMS) gradient of 3 × 10^–5^ Eh/au, max gradient of 10^–4^ Eh/au, RMS displacement of 6 × 10^–4^, and max displacement of 1 × 10^–3^ au).

The density functionals used were BP86,^80,81^ B97-D3^82^ (uses D3BJ), TPSS,^83^ TPSSh,^83,84^ BLYP,^80,85^ B3LYP,^80,85,86^ B3LYP*,^87,88^ PBE,^85^ PBE0,^89,90^ M06,^91^ M06-2X,^91^ BHLYP,^92^ CAM-B3LYP,^93^ ωB97M-D3BJ,^94,95^ ωB97X-D3BJ,^94,96^ and r^2^SCAN.^97^ As ωB97M-D3BJ and ωB97X-D3BJ are based on their parent ωB97M-V and ωB97X-V functionals but have been reparameterized for the D3BJ correction,^94^ we include D3BJ as a label (which also distinguishes the functional from the different ωB97X and ωB97X-D3 functionals^98^). The D3 dispersion correction with Becke–Johnson damping, DFT-D3BJ,^99,100^ was used for all functionals except for the Minnesota (M06 and M06-2X) functionals, where the older zero-damping^99^ version was used. The scalar relativistic zeroth order regular approximation (ZORA)^101,102^ was used in all calculations described in the Density Functional Comparison of the FeMoD11 and FeCSD5 Test Sets, Correlation between Bridging Metal–Ligand Bond Lengths and Metal–Metal Distance in FeMoD11, and Correlation between Fe–S Bond Covalency and Fe–Fe Distance sections. The ZORA calculations utilized the one-center approximation, and a relativistically recontracted triple-ζ def2 Ahlrichs basis set^103,104^ (ZORA-def2-TZVP keyword in ORCA) was used on all atoms except for Mo, where a ZORA-recontracted all-electron Ahlrichs basis set, TZVPPAlls,^104,105^ was used. The Basis Set, Relativistic, and Environmental Effects section describes calculations using other basis sets and relativistic approximations. Fine Lebedev angular integration grids were used for the exchange–correlation integrals (Grid5/Finalgrid6 keywords in ORCA), whereas for M06 and M06-2X, even tighter grids were used (Grid7 for M06 and M06-2X). The Split-RI-J approximation was used for Coulomb integrals in nonhybrid calculations, while the RIJCOSX^106,107^ approximation was used for Coulomb and Exchange integrals (GridX7 grid for COSX). Calculations using the r^2^SCAN functional were performed using ORCA version 5.0.0, using the tight grid settings (defgrid3 keyword) and using the libXC library to define the functional.^108^ A decontracted Coulomb auxiliary basis set by Weigend^103^ was used (SARC/J keyword) with the RIJ and RIJCOSX approximations. A polarizable continuum model (conductor-like polarizable continuum model (CPCM))^109^ including a Gaussian-charge scheme was included in all DFT calculations with a scaled van der Waals surface.^110,111^ We used the default vdW radii in ORCA with a 1.2 scaling factor as recommended in the implementation; this corresponds to scaled Bondi radii for the main group elements (C, N, O, P, S, and Cl), a radius of 1.32 Å for H (after scaling), and a radius of 2.4 Å (after scaling) for the heavy elements (here Fe and Mo). An infinite dielectric constant was used, as a crude mimic of a polar crystal environment and to stabilize the molecular anions that would otherwise have unbound electrons (see the Basis Set, Relativistic, and Environmental Effects section).

Antiferromagnetic broken-symmetry states of each spin-coupled dimer were located via the spin-flipping procedure implemented in ORCA. For the case of the Fe_A_(II)–Fe_B_(III) mixed-valence compounds in this study, we calculate a single broken-symmetry state with a localized Fe_A_(II)–Fe_B_(III) on either Fe_A_ or Fe_B_. As the complexes are symmetric, an isoenergetic broken-symmetry solution exists with a reversed oxidation state distribution. While these different solutions lead to distinct geometries, the Fe–Fe distance as well as the average bridging Fe–L distances are the same for both.

The FeMoco QM/MM model used here is the same as previously described,^22^ but for the reader’s convenience, we include the following short summary. QM/MM calculations are performed within Chemshell (version 3.7)^113,114^ using ORCA (version 4.2.1 and 5.0.0) for the QM part and DL_POLY^115^ for the MM part. The QM/MM model is spherical and contains 36 987 atoms with a QM region of 244 atoms (not counting link atoms terminating the QM–MM border). The QM theory level of the QM/MM calculations of FeMoco is similar to the QM calculations in this work except we use the ZORA-def2-TZVP basis set for Fe, S, Mo, and interstitial carbide, whereas ZORA-def2-SVP was used for all other atoms.

All figures of molecules presented herein are rendered using Visual Molecular Dynamics (VMD).^112^

## Results and Discussion

We will first introduce the test set of spin-coupled Fe–Fe and Mo–Fe dimers (FeMoD11) along with a test set of five closed-shell dimers (FeCSD5) and discuss the experimental reference data. In the Basis Set, Relativistic, and Environmental Effects section, we discuss the effect of basis sets, scalar relativistic approximation, and the polarizable continuum on the geometry of the spin-coupled [Fe_2_S_2_Cl_4_]^2–^ (**7**) as an example. In the Density Functional Comparison of the FeMoD11 and FeCSD5 Test Sets section, we discuss the results of the functional dependence of the geometry of the complexes of FeMoD11 and FeCSD5. The Correlation between Bridging Metal–Ligand Bond Lengths and Metal–Metal Distance in FeMoD11 section analyzes the correlation between bridging ligand–metal bond length and metal–metal distance for the spin-coupled systems compared to closed-shell systems. Finally, in the Correlation between Fe–S Bond Covalency and Fe–Fe Distance section, we discuss in detail the electronic structure of a representative system (**7**) from FeMoD11 and analyze how the covalency of the bridging ligand–metal bond affects the metal–metal distance.

### FeMoD11 Test Set

**Figure 2:**
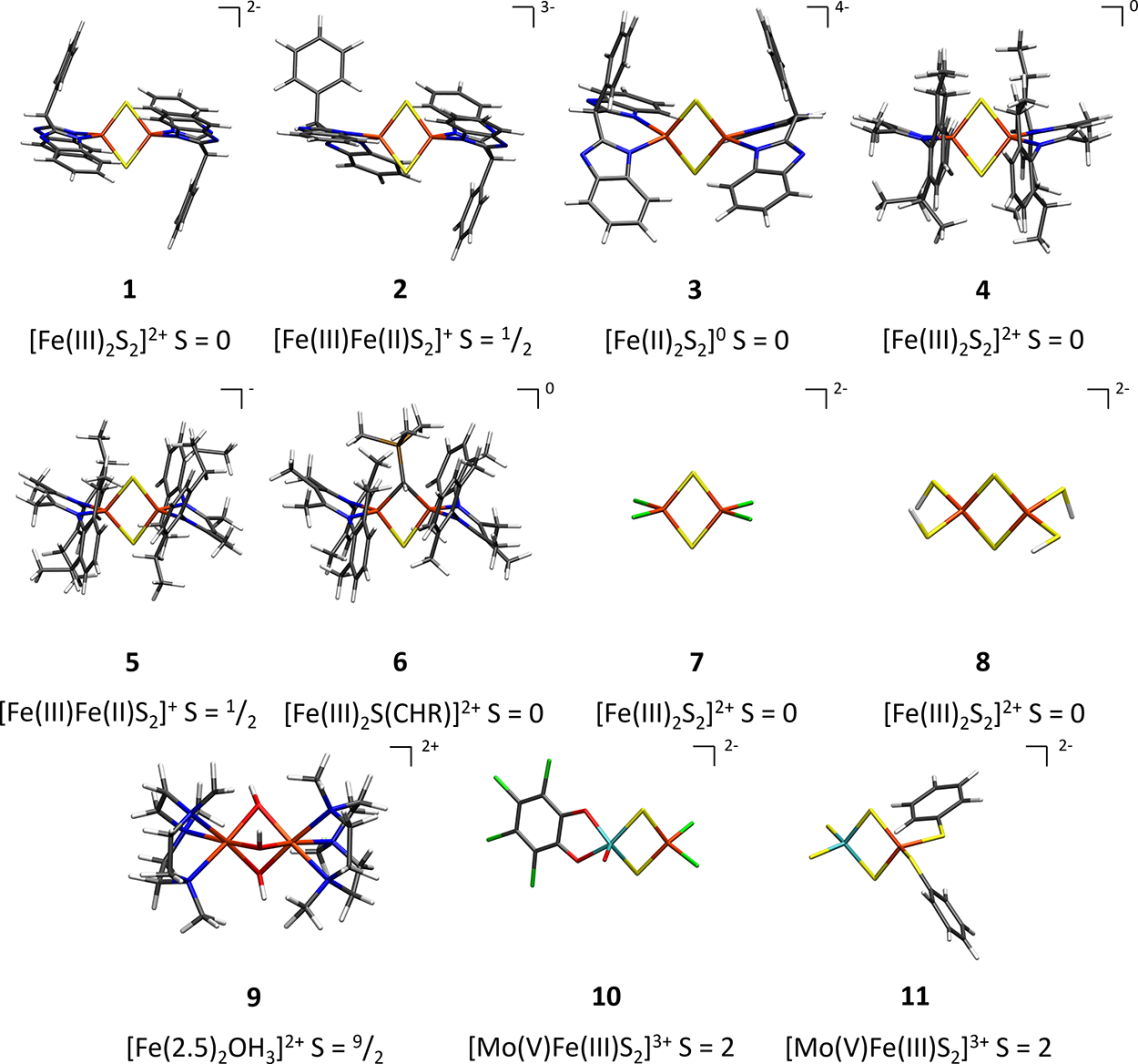
FeMoD11 test set of spin-coupled Fe–Fe and Fe–Mo dimers. The local oxidation state of each Fe ion is indicated as well as the charge of the core structure, the total spin, and the total charge of the complex.

The FeMoD11 test set, defined in Table 1 and Figure 2, contains 11 spin-coupled Fe–Fe or Mo–Fe dimers. Ten of these systems feature an [M–Fe–S–R] (M = Fe, Mo and R = S, C) diamond core structure with different terminal ligands in tetrahedral coordination geometries (except for one five-coordinate Mo geometry in **10**). These dimeric complexes were chosen as their core geometries [2Fe–2S], [2Fe–S–C], and [Mo–Fe–2S] can all be found in FeMoco; hence, both their molecular structure and spin-coupled electronic structure bear some resemblance to the enzyme cofactor of Mo-nitrogenase. Additionally, we include a [2Fe-3OH] complex (**9**) with octahedral iron coordination, which is a rare example of a complex with a mixed-valence spin-delocalized *S* = 9/2 ground state^116−118^ (mixed-valence delocalization being also a feature of polynuclear iron–sulfur clusters like FeMoco). Overall, the complexes feature the common Fe oxidation states that are observed for iron–sulfur systems: Fe(II) and Fe(III), with the mixed-valence complexes in the test set featuring either spin-localization (**2** and **5**) or delocalization (**9**). We note in this context the recent discovery of highly unusual mixed-valence Fe(II)–Fe(III) selenium/tellurium bridged dimers with *S* = 3/2 ground states.^119^

**Table 1:** FeMoD11 Test Set^a^

complex	CSD ID	*S*	charge^b^	M ox.^c^	bridging L	terminal L	counterion	temp (K)	R (%)^d^	M–Fe (Å)^e^
**1**	UZOHEX	0	2–	2 × Fe^3+^	2 × S^2–^	2 × bis(benzimidazolato)^2– ^	2 × (NEt_4_)^+^	133	3.21	2.702
**2**	UZOHIB	1/2	3–	Fe^2+^, Fe^3+^	2 × S^2–^	2 × bis(benzimidazolato)^2– ^	3 × (NEt_4_)^+ ^g	133	5.72	2.686
**3**	CEWTIJ	0	4–	2 × Fe^2+^	2 × S^2–^	2 × bis(benzimidazolato)^2– ^	4 × (NEt_4_)^+^	100	6.55	2.748
**4**	MUWQUS	0	0	2 × Fe^3+^	2 × S^2–^	2 × nacnac	N/A	150	3.63	2.679
**5**	MUWRED	1/2	1–	Fe^2+^, Fe^3+^	2 × S^2–^	2 × nacnac	1 × (NBut_4_)^+^	150	2.75	2.689
**6**	FUQYUO	0	0	2 × Fe^3+^	S^2–^, CHSi(CH_3_)_3_^2–^	2 × nacnac	N/A	93	3.07	2.603
**7**	EAFESD	0	2–	2 × Fe^3+^	2 × S^2–^	4 × Cl^–^	2 × (NEt_4_)^+^	295	3.60	2.714
**8**	XUQVAI	0	2–	2 × Fe^3+^	2 × S^2–^	4 × SH^–^	2 × (Ph_3_P)N_2_^+^	100	4.06	2.695
**9**	VADDEJ01	9/2	2+	2 × Fe^2.5+^	3 × OH^–^	2 ×tmtacn^f^	(ClO_4_)^−^	193	10.8	2.508
**10**	LAJPAN	2	2–	Fe^3+^, Mo^5+^	2 × S^2–^	Cl_4_cat^2–^, O^2–^, 2 × Cl^–^	2 × (NEt_4_)^+^	295	6.26	2.756
**11**	EAPTFM01	2	2–	Fe^3+^, Mo^5+^	2 × S^2–^	2 × SPh^–^, 2 × S^2–^	2 × (NEt_4_)^+^	295	4.9	2.765

a The table includes information on spin, charge, oxidation state, bridging ligands, counterions, as well as crystallographic data: crystallized counterion, X-ray diffraction temperature, *R*-factor, and metal–metal distance.

b Total charge of the complex.

c Local oxidation state of the Fe/Mo ions.

d The conventional residual factor.

e M = Fe or Mo.

f 1,4,7-Trimethyl-1,4,7-triazononane.

g Cobaltocene is additionally present in the crystal structure.

Our choice to focus on spin-coupled dimers rather than larger multinuclear clusters is motivated by the simpler electronic structure in dimers than in trimers or tetramers, where a single electronic state (usually the low-spin antiferromagnetic state) should generally be well separated from other states, which is not necessarily the case for multinuclear clusters where complex spin couplings including, e.g., spin-canting effects and double exchange, can lead to a highly complex spin ladder. As will be shown, the molecular and electronic structures of these simple dimer compounds are still highly relevant to the much more complex FeMoco cluster as discussed in the Density Functional Comparison of the FeMoD11 and FeCSD5 Test Sets and Correlation between Bridging Metal–Ligand Bond Lengths and Metal–Metal Distance in FeMoD11 sections.

Complexes **1**–**3**^120,121^ are [2Fe–2S] systems from the Meyer group featuring the bis(benzimidazolato) ligand; this was the first set of dimers that was synthesized in all three redox states (2Fe(III), Fe(III)Fe(II), and 2Fe(II)) characterized by X-ray crystallography, Mössbauer spectroscopy, superconducting quantum interference device (SQUID), and cyclic voltammetry. The overall quality of the X-ray structures is good, with complexes **1**, **2**, and **3** having *R*-factors of 3.21, 5.72 (also 7.22), and 6.5%, respectively. Two X-ray structures are available for complex **2**, with different counterions. The structure containing both an NEt_4_^+^ counterion and cobaltocene (CSD code: UZOHIB) with *r*(Fe–Fe) = 2.686 Å was included in our test set as it has a lower *R*-factor (5.72 vs 7.22%) than the other structure with only NEt_4_^+^ counterion (CSD code: CEWTOP (*r*(Fe–Fe) = 2.727 Å)).

Complexes **4** and **5**(122) are [2Fe–2S] complexes from the Driess group, with β-diketiminato (nacnac) ligands and in two different redox states (2Fe(III) and Fe(III)Fe(II)). The X-ray structures of **4** and **5** are of high quality with *R*-factors of 3.63 and 2.75%, respectively.

Complex **6**(123) has a [2Fe–S–C] core and contains one bridging alkylidene group and one bridging sulfide (instead of two sulfides) with terminal β-diketiminato ligands on the Fe ions, with Fe(III) oxidation states and an *R*-factor of 3.07%.

Complexes **7**(124) and **8**(125) are comparatively small and without bulky ligands (complex **7** has terminal chloro ligands and complex **8** has terminal thiolate ligands). The X-ray structures are of high quality with *R*-factors of 3.6 and 4.06%, respectively. Both complexes feature the 2Fe(III) redox state.

Complex **9**(117) by Wieghardt and co-workers is different from the previously discussed complexes **1**–**8** as it contains three hydroxo bridging ligands in a [2Fe–3OH] core with octahedral Fe ions. Although not an iron–sulfur system (and lacking a diamond core), it is of interest due to being a rare case of a mixed-valence system with a ground-state spin of *S* = 9/2. The electronic structure of this complex has been thoroughly characterized^116−118^ and is interpreted as containing complete delocalization of the minority-spin electron, resulting in a physical oxidation state description of 2Fe(2.5). The terminal ligands are 1,4,7-trimethyl-1,4,7-triazonane (tmtacn). The X-ray structure has a relatively high *R*-factor of 10.8%. However, as extended X-ray absorption fine structure (EXAFS) measurements indicate an *r*(Fe–Fe) distance of 2.50 ± 0.01 Å, which is in good agreement with the X-ray structure (*r*(Fe–Fe) = 2.508 Å), we consider the X-ray structure nonetheless reliable (at the very least the Fe–Fe distance) and include it in our benchmarking. We note in the context of **9** that another complex from Wieghardt and co-workers with a [2Cr–3OH] core^126^ and tmtacn ligands has been the subject of recent discussion in the literature. There is an ongoing debate whether the antiferromagnetism results from the direct overlap of d-orbitals or from superexchange.^127−129^

Complexes **10**(130) and **11**(131) are [MoFe–2S] systems and feature Mo–Fe interactions that resemble Mo–Fe interactions proposed in FeMoco. In both complexes, the molybdenum has been proposed to be in a Mo(V) oxidation state, while iron is in a Fe(III) oxidation state, although ^57^Fe Mössbauer experiments suggest complicated spin delocalization effects. While FeMoco features an unusual Mo(III) oxidation state,^9^ we note that similar spin delocalization in the Mo–Fe interactions has been proposed for FeMoco. Complexes **10** and **11** have good *R* values, 6.26 and 4.9%, respectively. Complex **10** has two chloro ligands connected to iron, whereas the molybdenum is ligated to tetrachlorocatecolate and an oxo ligand. Complex **11** has two thiophenyl ligands ligated to iron, whereas the molybdenum is ligated to two sulfurs.

**Figure 3:**
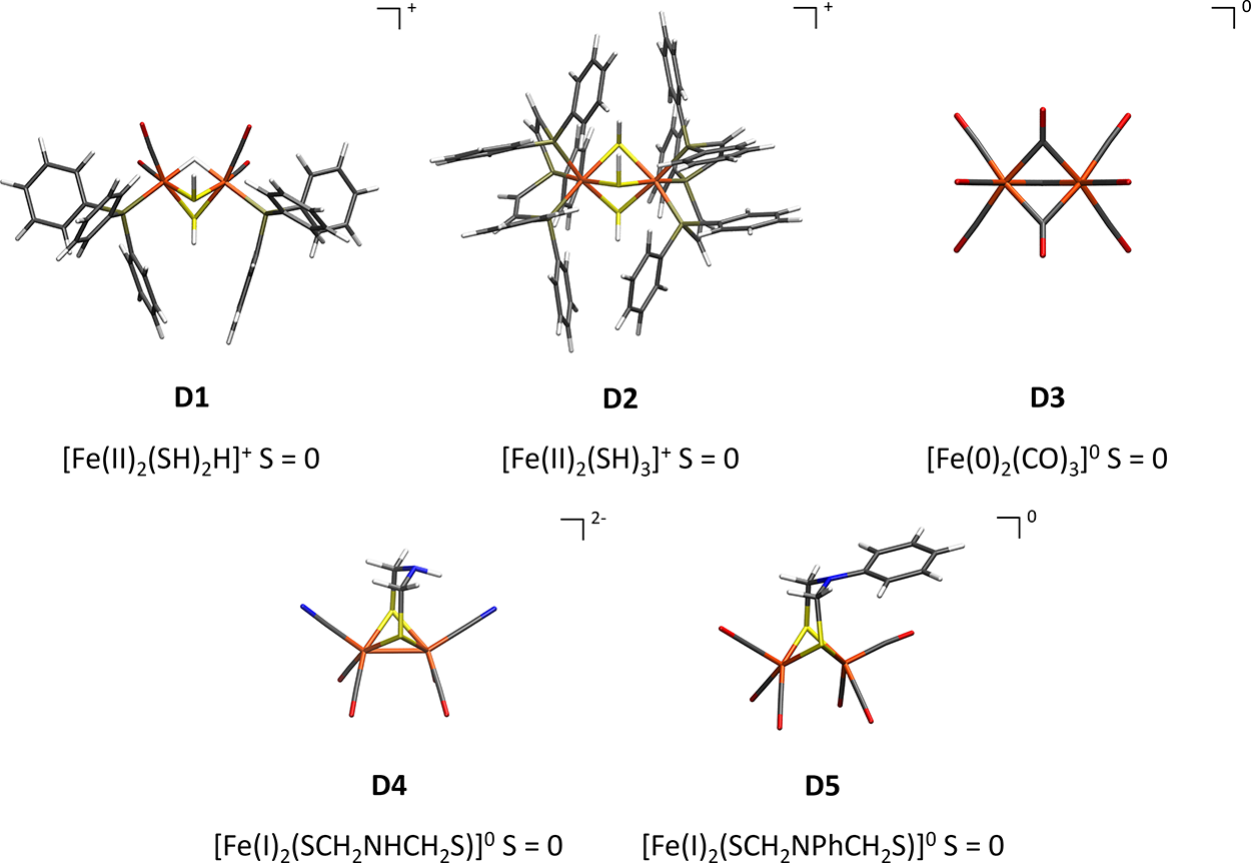
FeCSD5 test set of closed-shell Fe–Fe dimers. The local oxidation state of each Fe ion is indicated as well as the charge of the core structure, the total spin, and the total charge of the complex.

For comparison to the spin-coupled dimers in the FeMoD11 test set, we created another test set of five diamagnetic closed-shell complex dimers with local low-spin irons (in oxidation states Fe(II), Fe(I), and Fe(0)) and a diamagnetic ground state of *S* = 0, which we will term here FeCSD5 (Fe closed-shell dimers); see Figure 3. Complexes **D1**(132) and **D2**(133) contain both locally low-spin irons in a Fe(II) oxidation state, whereas the former contains two bridging SH^–^ ligands and a single bridging hydride and the latter three bridging SH^–^ ligands. **D1** has two CO and one triphenylphosphine ligands on each of its irons, whereas **D2** is terminally ligated with bis[2-(diphenylphosphino)ethyl]phenylphosphine on each of the irons. Complex **D3**(134) contains irons in a Fe(0) oxidation state and with three bridging carbonyl ligands and three-terminal carbonyl ligands on each iron. Complexes **D4**(135) and **D5**(135) are hydrogenase model complexes with irons in the 2Fe(I) oxidation state and a Fe–Fe σ-bond between the two irons. **D4** has a bridging S(CH_2_NHCH_2_)S^2–^ ligand, four CO terminal ligands, and two extra CN terminal ligands. **D5** has S(CH_2_N(Ph)CH_2_)S^2–^ bridging ligand and six CO terminal ligands.

**Table 2:** FeSCD5 Test Set^a^

complex	CSD ID	*S*	charge^b^	Fe ox.^c^	bridging L	terminal L	counterion	temp (K)	*R* (%)^d^	Fe–Fe (Å)
**D1**	NOBXUA	0	1+	2 × Fe^2+^	H^–^, 2 × SH^–^	4 × CO, 2 × PPh_3_	1 × (BAr^F^_4_)^−^	100	4.06	2.589
**D2**	PEPSFE	0	1+	2 × Fe^2+^	3 × SH^–^	2 × PPP^e^	(ClO_4_)^−^	295	3.63	3.192
**D3**	FUZGAI	0	0	2 × Fe(0)	3 × CO	6 × CO	N/A	295	4	2.523
**D4**	YOBSEN	0	2–	2 × Fe^+^	S(CH_2_NHCH_2_)S^2–^	2 × CN^–^, 4 × CO	2 × (NEt_4_)^+^	293	2.96	2.509
**D5**	YOBVEQ	0	0	2 × Fe^+^	S(CH_2_N(Ph)CH_2_)S^2–^	6 × CO	N/A	193	2.39	2.505

a The table includes information on spin, charge, oxidation state, bridging and terminal ligands (L), counterions, as well as crystallographic data: crystallized counterion, X-ray diffraction temperature, *R*-factor, and metal–metal distance.

b Total charge of the complex.

c Local oxidation state of each of the Fe ions.

d The conventional *R*-factor.

e Bis[2-(diphenylphosphino)ethyl]phenylphosphine.

The spin-coupled Fe–Fe dimers in FeMoD11 feature Fe–Fe distances that range from 2.508 to 2.748 Å. The delocalized mixed-valence compound **9** has the shortest Fe–Fe distance (2.508 Å), likely due to both the light bridging ligands (OH) and having a direct d–d interaction. Complex **6** has a short distance of 2.603 Å, due to a bridging carbon ligand in addition to the sulfide. Complexes **1**–**5** and **7**–**8** all feature the same [2Fe–2S] diamond core and the Fe–Fe distances from 2.686 to 2.748 Å. These distances seem to vary according to both the bulkiness of the terminal ligands and the metal oxidation states. Complexes **1**–**3** have the same ligand framework (bis(benzimidazolato) and the Fe–Fe distance changes somewhat nonintuitively from 2.702 Å (all-ferric) via 2.686 Å (mixed-valence) to 2.748 Å (all-ferrous). We note, however, the existence of another X-ray structure for the mixed-valence compound with a 2.727 Å distance (instead of 2.686 Å). Complex **3** is the only all-ferrous complex and has the longest Fe–Fe distance. Complexes **4** and **5** also feature the same ligand framework (nacnac) with different redox states, and a small 0.01 Å increase in Fe–Fe distance is observed upon going from all-ferric to mixed-valence. The nacnac ligand in **4** and **5** is the bulkiest ligand in the test set. Comparing the all-ferric complexes (**1**, **4**, **7**, **8**), we note that **4** has the shortest Fe–Fe distance (2.679 Å) while the least bulky complex **7** has the longest (2.714 Å). This may indicate the presence of a stabilizing dispersion effect between ligands that brings the metal ions closer together. Finally, we also note that the total charge may also be a factor in these comparisons, with complex **4** being neutral, while complexes **1**, **7**, and **8** are dianionic.

To summarize, the Fe–Fe distances in these complexes seem to vary according to the nature of the bridging ligand (largest effect), oxidation state, nature of terminal ligands, and possibly due to differing counterions and total complex charge.

In comparison, the **D1**–**D5** diamagnetic complexes in FeCSD5 in Table 2 and Figure 3 feature mostly shorter Fe–Fe distances (**D1**, **D3**, **D4**, **D5**) except for **D2** with a relatively long Fe–Fe distance of 3.192 Å. The short Fe–Fe distances in **D4** and **D5** are a consequence of a formal Fe–Fe σ-bond. Complex **D3** features a rather short Fe–Fe distance of 2.523 Å and was originally proposed to feature a Fe–Fe bond, but the short Fe–Fe distance is nowadays interpreted as arising from favorable covalent bridging Fe–CO–Fe interactions.^136^ Complex **D1** also features a rather short Fe–Fe distance, most likely due to the covalent bridging Fe–H–Fe bond. In comparison, complex **D2** features a very long Fe–Fe distance, apparently due to the three bridging thiol groups. The test set of **D1**–**D5** was designed to include Fe–Fe dimer complexes that lack a local high-spin electronic structure or spin coupling (no unpaired or spin-coupled electrons), in contrast to FeMoD11.

### Basis Set, Relativistic, and Environmental Effects

**Figure 4:**
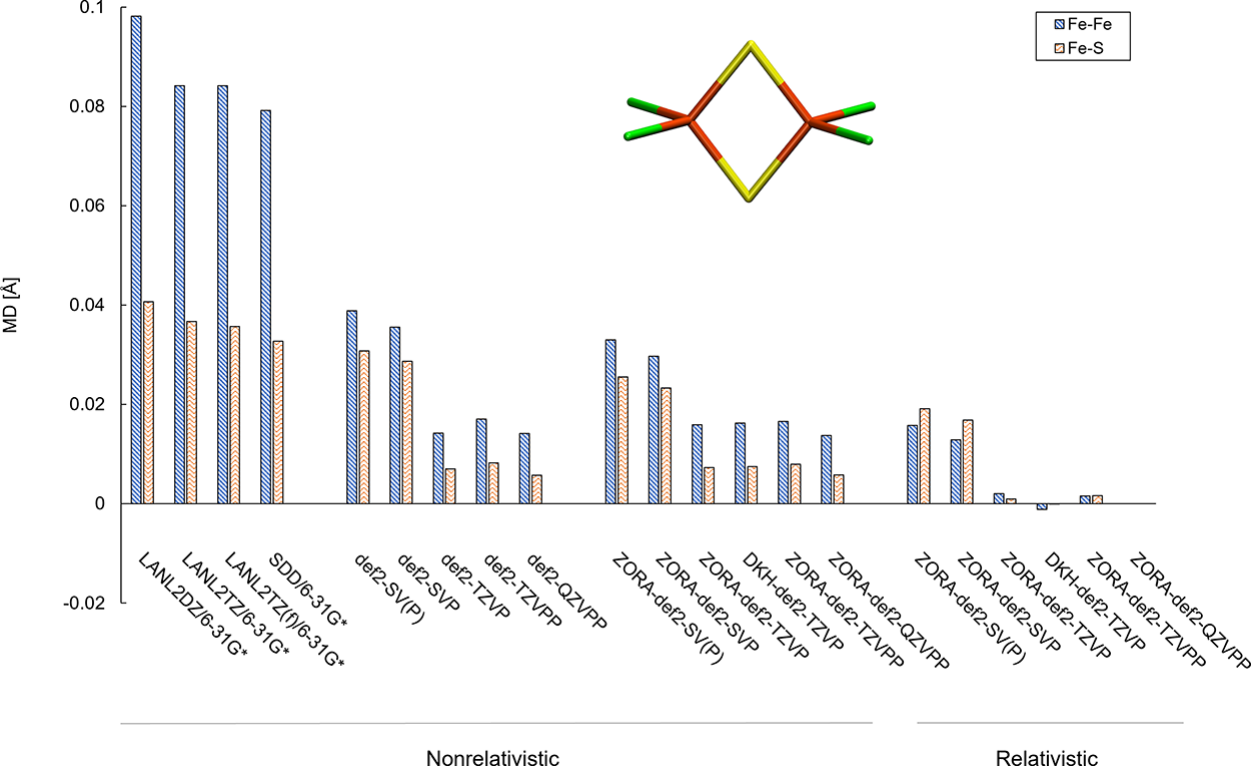
Mean deviations (MDs) of Fe–S bond lengths and Fe–Fe distance of complex **7** ([Fe_2_S_2_Cl_4_]^2–^) using ECP-basis combinations (LANL2 or SDD on Fe with 6-31G* on S and Cl) or all-electron basis sets, with and without a scalar relativistic ZORA or Douglas–Kroll–Hess (DKH) Hamiltonian. Deviations are relative to the largest all-electron relativistic ZORA-def2-QZVPP reference (*r*(Fe–Fe) = 2.690 Å and *r*_ave_(Fe–S) = 2.202 Å). The TPSSh functional was used with a CPCM(ε = ∞) continuum model included in all calculations.

Before discussing the density functional dependency for the FeMoD11 and FeCSD5 test sets, it is important to assess basis set effects as well as scalar relativistic effects that may affect such functional comparisons. The basis set dependency for iron–sulfur systems has previously been discussed by Szilagyi et al.,^75^ who found that both the geometry and spin density distribution were quite sensitive to the basis set size. We study here the basis set effects on the geometry of complex **7** as representative of the [2Fe–2S] core that is present in most compounds in FeMoD11. Figure 4 shows the deviation of both the Fe–Fe distance and the average Fe–S bond lengths for various basis sets, using the TPSSh hybrid density functional. The reference values are obtained using the large relativistically recontracted ZORA-def2-QZVPP basis set^103,104^ (with the ZORA scalar relativistic Hamiltonian included), which we estimate should be close to the DFT basis set limit. Overall, we find that the basis set errors are highly systematic for Fe–Fe and Fe–S distances, resulting consistently in overestimation with respect to (w.r.t.) the relativistic def2-QZVPP reference. Beginning with the ZORA relativistic results (employing the ZORA scalar relativistic Hamiltonian and the ZORA-recontracted ZORA-def2-XVP basis sets), we see that the results systematically approach the basis set limit. The basis set error is moderate for the double-ζ basis sets, ZORA-def2-SV(P) and ZORA-def2-SVP (+0.016/+0.013 Å for Fe–Fe and +0.019/+0.013 Å for Fe–S) while practically converged at the triple-ζ level, ZORA-def2-TZVP and ZORA-def2-TZVPP (+0.002/+0.002 Å for Fe–Fe and +0.001/0.001 Å for Fe–S). These results suggest that geometries of spin-coupled iron–sulfur compounds may generally be converged with a well-polarized triple-ζ basis set level.

In order to evaluate the effect of using a different scalar relativistic Hamiltonian approximation, we additionally obtained results at the second-order Douglas–Kroll–Hess (DKH) level with DKH-recontracted def2 basis sets.^104^ We obtain very similar results for *r*(Fe–Fe) with the DKH-TPSSh/DKH-def2-TZVP and the ZORA-TPSSh/ZORA-def2-TZVP levels of theory, 2.689 and 2.691 Å, respectively. In the case of *r*(Fe–S_ave_), it is 2.203 and 2.202 Å, respectively. These results indicate that the ZORA and DKH approximations account equally well for the scalar relativistic geometric effect on this system.

Looking at the nonrelativistic results, we compare the nonrelativistic all-electron Ahlrichs def2 family^103^ with respect to the ZORA/ZORA-def2-QZVPP reference. A systematic decrease in deviations with increased ζ-level of the basis set is evident; however, even at the def2-QZVPP level, an error remains, suggesting that the remaining deviation (+0.014 Å for Fe–Fe and +0.006 Å for Fe–S) arises due to a relativistic effect missing in the nonrelativistic calculations. This is further evidenced by the almost identical behavior of the results employing the relativistically recontracted basis sets (ZORA-def2-XVP) but without the ZORA Hamiltonian. Comparing the nonrelativistic def2-SV(P) basis set that has, for example, been employed in FeMoco research,^137^ we find that this results in a combined basis-set-error + lack-of-relativity error that amounts to +0.039 Å for Fe–Fe and +0.031 Å for Fe–S.

There are considerably larger errors associated with using common effective-core-potential/valence-basis protocols such as LANL2DZ/6-31G* or SDD/6-31G*, approximately 2 times larger error than the error from the smallest all-electron basis set (def2-SV(P)). Using the all-electron 6-31G* basis set^138,139^ on S and Cl and LANL2DZ, LANL2TZ, or LANL2TZ(f) on Fe (with the associated LANL2 ECP)^140^ results in relatively large basis set errors for Fe–Fe distances (0.084–0.098 Å) and Fe–S bond lengths (0.036–0.041 Å). This basis set + ECP combination can thus not be recommended for describing iron–sulfur chemistry, despite its use in mechanistic studies of the nitrogenase iron–molybdenum cofactor in recent studies.^27^ Results employing the SDD ECP + basis^141,142^ set on Fe and 6-31G* on S and Cl give similarly poor results as well, with errors of +0.079 Å for Fe–Fe and +0.033 Å for Fe–S. These large errors most likely arise due to the effective core potential on Fe, although this was not further investigated. We note that this agrees with previous studies that found considerable errors for 3d transition-metal complexes when ECPs were used.^72,143^

Overall, we find that the basis set effects for complex **7** are not overly large for modern all-electron basis sets (such as the Ahlrichs def2 family) and that the polarized ZORA-def2-TZVP basis set has an acceptably low basis set error. The scalar relativistic effects on the geometry (+0.016 Å for Fe–Fe and +0.007 Å for Fe–S) are small but worth accounting for, as the computational cost associated with the relativistic integrals is very small. The ZORA-def2-TZVP basis including the ZORA Hamiltonian will hence be used throughout this study. We note that the use of a valence-basis + ECP for a 4d transition metal (such as Mo) is likely more justified than for a 3d transition metal (and may account well for scalar relativistic effects); however, the use of ECPs on Mo for the Mo complexes in this work was not investigated and the all-electron ZORA approach was used throughout (using a ZORA-recontracted all-electron triple-ζ basis set; see the Computational Details section).

Complex **7** is an anion with a charge of 2–. As dianions are typically not stable in the gas phase, a conductor-like polarizable continuum model (CPCM) was used to screen the high negative charge, and this approach has been used in all calculations of the complexes in this study (whether cationic, anionic, or neutral). CPCM acts as an approximation to the polar crystal environment by describing it as a homogeneous polarizable continuum characterized by a global dielectric constant. While this continuum approach cannot account for specific crystal effects such as counterions, hydrogen bonding, intermolecular dispersion within the crystal, it should be generally preferable to calculating charged molecules in the vacuum. Table 3 shows the effect of including the CPCM model with varying dielectric constant on the structure of complex **7**. A vacuum calculation (ε = 1) of **7** gives eight unbound electrons (occupied molecular orbitals (MOs) with positive energies), confirming that the dianion is not stable in the gas phase, and this appears to lead to overestimated Fe–Fe and Fe–L distances compared to the crystal structure. Including the CPCM model with ε = 4, however, stabilizes the unstable MOs and leads to geometric bond contractions. Further increasing the dielectric constant leads to slight geometric changes that converge at ε = 10, with further negligible changes up to ε = ∞. As the dielectric constant cannot easily be determined for different crystals, we chose to use CPCM(ε = ∞) for all DFT calculations in this work.

**Table 3:** Effect of Varying the CPCM Dielectric Constant on the Structural Parameters of **7** at the ZORA-TPSSh/ZORA-def2-TZVP Level^a^

dielectric constant ε	1	4	10	20	40	80	∞	X-ray
Fe1–Fe2 (Å)	2.740	2.703	2.696	2.693	2.692	2.692	2.692	2.714
Fe1–Cl1 (Å)	2.280	2.262	2.258	2.257	2.257	2.256	2.256	2.244
Fe1–Cl3 (Å)	2.280	2.262	2.258	2.257	2.256	2.256	2.255	2.256
Fe1–S1 (Å)	2.211	2.205	2.204	2.203	2.203	2.203	2.203	2.201
Fe1–S2 (Å)	2.211	2.204	2.203	2.202	2.202	2.202	2.202	2.198
Fe2–Cl2 (Å)	2.280	2.262	2.258	2.257	2.256	2.256	2.255	2.244
Fe2–Cl4 (Å)	2.280	2.262	2.258	2.257	2.256	2.256	2.256	2.256
Fe2–S1 (Å)	2.211	2.204	2.203	2.202	2.202	2.202	2.202	2.198
Fe2–S2 (Å)	2.211	2.205	2.204	2.203	2.203	2.203	2.203	2.20
average Fe–S (Å)	2.211	2.205	2.204	2.203	2.203	2.203	2.203	2.199
average Fe–Cl (Å)	2.280	2.262	2.258	2.257	2.256	2.256	2.256	2.250
no. of unbound electrons	8	0	0	0	0	0	0	

a Also shown is the number of unbound electrons (occupied MOs with positive energies) in each calculation. Structural parameters from the X-ray structure are shown for comparison.

### Density Functional Comparison of the FeMoD11 and FeCSD5 Test Sets

We now turn to the results of the functional comparison for both the FeMoD11 and the FeCSD5 test sets. The focus of our comparison is on the Fe–Fe/Mo–Fe distance, both because that distance turns out to be highly sensitive to the theory level and because the positions of the heaviest atoms from an X-ray crystal structure should have lower structural uncertainties than lighter atoms.

**Figure 5:**
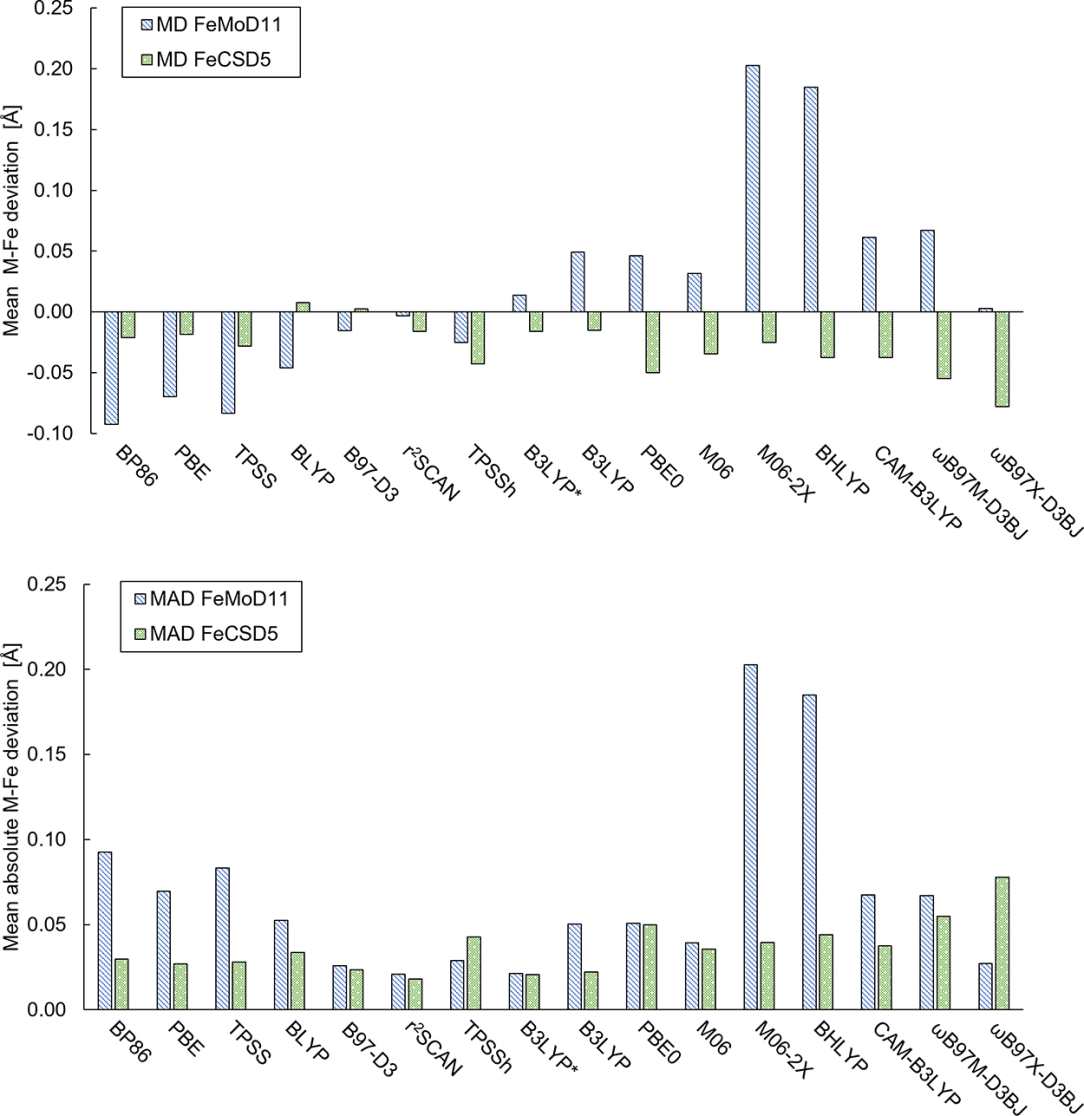
Top: mean deviation (MD) of M–Fe (M = Mo, Fe) distances for optimized structures of FeMoD11 and FeCSD5 with different functionals w.r.t. the X-ray structure distances. Bottom: the corresponding mean absolute deviation (MAD). All calculations use a ZORA scalar relativistic Hamiltonian, the relativistically recontracted ZORA-def2-TZVP basis set, a D3BJ (except D3 for M06 and M06-2X) dispersion correction, and CPCM(ε = ∞).

Figure 5 shows the mean deviations (MD) and mean absolute deviations (MAD) for both the FeMoD11 and FeCSD5 test sets for all density functionals considered in our study. The first thing to note is the different functional trends for the two test sets. While all density functionals (BLYP and B97-D3 being the exceptions) systematically underestimate the Fe–Fe distance in the FeCSD5 test set (see Figure 5, top) with no clear trend between hybrid and nonhybrid functionals, there is much greater variation in the data for the FeMoD11 test set.

The data clearly shows that the spin-coupled dimers of FeMoD11 are highly sensitive to the amount of exact exchange in the functional. The common nonhybrid functionals, BP86, PBE, and TPSS underestimate the Fe–Fe/Mo–Fe distance on average, giving MDs of −0.093, −0.070, and −0.083 Å (MADs of 0.093, 0.070, and 0.083 Å), respectively. The BLYP functional also underestimates (MD/MAD = −0.046/0.053 Å), although not as much as BP86, PBE, and TPSS. On the other hand, hybrid functionals with a large amount of exact exchange (≥50%) overestimate the Fe–Fe/Mo–Fe distance considerably, with BHLYP (a hybrid generalized gradient approximation (GGA) with 50% exact exchange) and M06-2X (a hybrid meta-GGA with 54% exact exchange) yielding MDs of +0.185 and +0.203 Å (MADs of 0.185 and 0.203 Å), respectively.

TPSSh (a hybrid meta-GGA functional with 10% exact exchange) is the only hybrid functional to underestimate the average Fe–Fe/Mo–Fe distance and gives an MD of −0.023 Å (MAD 0.029 Å). B3LYP* (a hybrid GGA functional with 15% exact exchange, proposed by Reiher and co-workers^87,88^) also yields good Fe–Fe/Mo–Fe distances on average, with MD = +0.014 Å (MAD 0.021 Å) while slightly overestimating the Fe–Fe/Mo–Fe distance. The functionals with 20–28% exact exchange: B3LYP (a hybrid GGA with 20% exact exchange), PBE0 (hybrid GGA with 25% exact exchange), and M06 (a hybrid GGA with 26% exact exchange) give overall similar structures, overestimating the Fe–Fe distance in general and giving MD values of 0.049, 0.046, and 0.032 Å (MAD = 0.050, 0.051, and 0.039 Å), respectively.

The range-separated hybrid functionals, i.e., CAM-B3LYP, ωB97M-D3BJ, and ωB97X-D3BJ, appear not to offer clear advantages over the regular hybrid functionals. For Fe–Fe/Mo–Fe distances, CAM-B3LYP gives worse deviations for both spin-coupled (and diamagnetic complexes) than its parent B3LYP functional with MD = +0.061 Å (MAD 0.067 Å) and the recent ωB97M-D3BJ functional (found to be highly accurate for main group thermochemistry)^94,144^ offers no improvement either, with MD of +0.067 Å (MAD = 0.067 Å). The ωB97X-D3BJ functional, however, appears much more promising for treating the spin-coupled systems, with MD = +0.003 Å and MAD = 0.027 Å.

Interestingly, the nonhybrid functionals r^2^SCAN and B97-D3 break the trend of systematic strong underestimation of the Fe–Fe/Mo–Fe distances with nonhybrid functionals for FeMoD11, being much closer to a mean deviation of 0 and give in fact among the best results for FeMoD11 (along with B3LYP*), according to the mean absolute deviations (MADs of 0.021 and 0.026 Å), respectively. This shows that in addition to the exact exchange component, the exchange and correlation functional components clearly play also a major role in describing these systems.

Within the FeMoD11 test set, there is some variance seen in the behavior of the functionals between the Fe–Fe dimers and Mo–Fe dimers (see Figure S7). PBE0 is considerably better for the Mo–Fe systems than for the Fe–Fe systems, with an MD of +0.010 Å (MAD 0.010 Å) for FeMoD11(**10**,**11**) in comparison to an MD of +0.050 Å (MAD 0.055 Å) for the Fe–Fe systems FeMoD11(**1**–**9**). In contrast, the ωB97X-D3BJ functional yields worse geometries for the Mo–Fe systems than for the Fe–Fe systems, where for the FeMoD11(**10**,**11**) the functional yields optimized geometries with an MD of −0.049 Å (MAD 0.049 Å) in comparison to the Fe–Fe dimers of FeMoD11(**1**–**9**) with an MD of +0.014 Å (MAD 0.022 Å). Additionally, the nonhybrid functionals, BLYP and B97-D3, do not underestimate the metal–metal distance as much for the Mo–Fe dimers (FeMoD11(**10**,**11**)) with an MD of −0.009 and −0.002 Å (MAD = 0.009 and 0.013 Å), respectively, in comparison to the Fe–Fe dimers (FeMoD11(**1–9**)), where BLYP and B97-D3 underestimate the Fe–Fe distance (not as much as BP86, PBE, and TPSS) with an MD of −0.054 and −0.018 Å (MAD = 0.062 and 0.029 Å) respectively.

In contrast to the FeMoD11 test set, almost all functionals underestimate on average the Fe–Fe distance of the closed-shell complexes in the FeCSD5 test set (BLYP and B97-D3 being curious exceptions), and there is no clear trend observed with an increase in exact exchange with hybrid functionals. The FeMoD11 test set thus clearly features complexes with a more sensitive electronic structure that results in a stronger variation of the resulting molecular geometries. Interestingly though, the range-separated hybrid ωB97X-D3BJ functional, which performed well for the FeMoD11 test set (third lowest MAD), yields the worst geometries of all functionals tested for the FeCSD5 set, with an MD of −0.078 Å (MAD 0.078 Å). All other functionals have MAD values from 0.018 to 0.055 Å. The best performing functional for FeCSD5 is r^2^SCAN with MAD = 0.018 Å.

**Figure 6:**
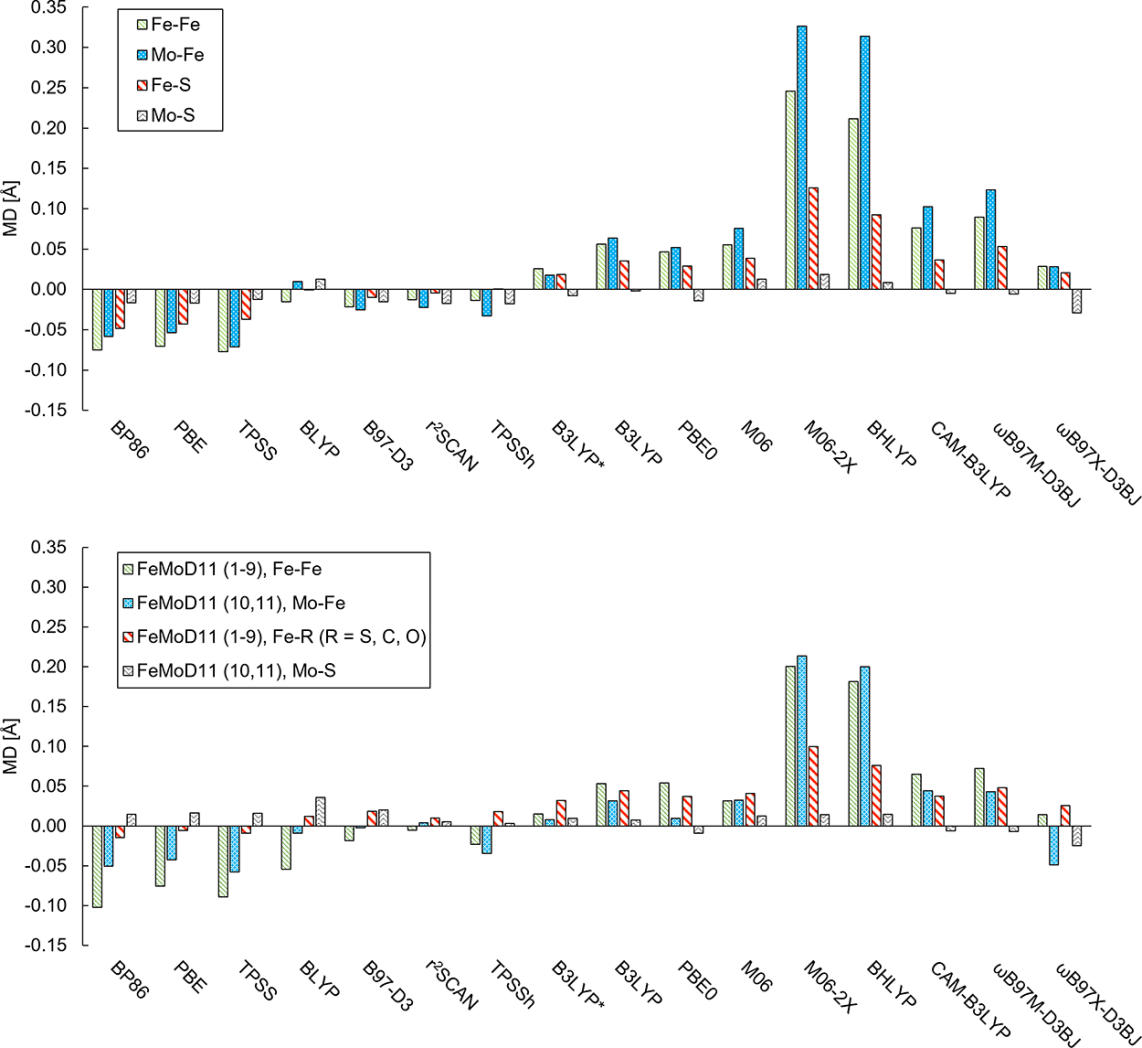
Top: mean deviations of Fe–Fe, Mo–Fe, Fe–S, and Mo–S distances from 244 QM-atom QM/MM calculations (deviations relative to the 1.0 Å crystal structure, PDB ID: 3U7Q(2)). Bottom: mean deviations of the Fe–Fe, Mo–Fe, Fe–S, and Mo–S distances in the FeMoD11 test set relative to each respective crystal structure. A plot of the corresponding mean absolute deviations is available in the Supporting Information (SI) as Figure S1.

The systematic underestimation of Fe–Fe/Mo–Fe distances by nonhybrid functionals BP86, PBE, and TPSS and overestimation of hybrid functionals such as B3LYP and M06-2X for the FeMoD11 test set of spin-coupled Fe–Fe and Fe–Mo dimers are not entirely surprising compared to our previous work on FeMoco of nitrogenase.^29^ In that work, density functionals were assessed on their ability to describe the resting-state geometry of the multimetal spin-coupled FeMoco at the QM/MM level by comparison to the high-resolution 1.0 Å X-ray crystal structure.^2^Figure 6 compares the mean deviations for Fe–Fe and Mo–Fe distances in FeMoco (calculated using a QM/MM model) to the analogous distances in the FeMoD11 test set with the same functionals. Despite some differences in the magnitudes of the errors for the FeMoD11 set compared to FeMoco, we clearly see the same trend of the errors for different functional classes, strongly implying that the errors are related to each other (most likely due to a related electronic structure as will be discussed). The data shows that Fe–Fe and Mo–Fe distances are underestimated with the nonhybrid functionals BP86, PBE, and TPSS (while r^2^SCAN, BLYP, and B97-D3 have MDs closer to zero), for both the FeMoD11 test set and FeMoco while they tend to be overestimated for global hybrid functionals and range-separated hybrid functionals. Overall, the trends in density functional errors for the FeMoD11 test set correlate well with the behavior to describe the Fe–Fe/Mo–Fe distances in FeMoco: functionals with low MAD values for FeMoD11 give low MAD values for FeMoco (BLYP being an exception).

The nonhybrid functionals that underestimate Fe–Fe/Mo–Fe distances in FeMoD11 (BP86, PBE, TPSS) do the same for FeMoco, while functionals with >20% exact exchange overestimate the distances (dramatically so if >50% exact exchange) for both FeMoD11 and FeMoco. The range-separated hybrids do not offer clear improvements (though ωB97X-D3BJ appears more promising).

Table 4 compares the overall functional statistics for FeMoD11, FeCSD5, and the FeMoco geometry. The best performing functionals for the M–M distances of the FeMoD11 test set (based on MAD values) are r^2^SCAN (0.021 Å), B3LYP* (0.021 Å), B97-D3 (0.026 Å), ωB97X-D3BJ (0.027 Å), and TPSSh (0.029 Å). For the FeCSD5 set, the best functionals according to MAD values are r^2^SCAN (0.018 Å), B3LYP* (0.021 Å), B3LYP (0.022 Å), and B97-D3 (0.023 Å). For the FeMoco system, the best performing functionals are r^2^SCAN (0.017 Å), TPSSh (0.020 Å), B97-D3 (0.024 Å), BLYP (0.024 Å), B3LYP* (0.026 Å), and ωB97X-D3BJ (0.028 Å).

**Table 4:** Mean Deviations (MD), Mean Absolute Deviations (MAD), Root-Mean-Square Deviations (RMSD)^a^ and Max Deviations (MAX) in Å for Metal–Metal and Metal-Ligand Distances of FeMoD11, FeCSD5, and FeMoco^b^^c^^d^^f^

	BP86	PBE	TPSS	B97-D3	BLYP	r^2^SCAN	TPSSh	B3LYP*	B3LYP	PBE0	M06	M06-2X	BHLYP	CAM-B3LYP	ωB97M-D3BJ	ωB97X-D3BJ
FeMoD11																
RMSD^a^	0.067	0.045	0.049	0.035	0.043	0.029	0.036	0.038	0.045	0.043	0.078	0.108	0.085	0.049	0.051	0.040
MD M–Fe^b^	–0.093	–0.070	–0.083	–0.015	–0.046	–0.003	–0.025	0.014	0.049	0.046	0.032	0.203	0.185	0.061	0.067	0.003
MAD M–Fe^b^	0.093	0.070	0.083	0.026	0.053	0.021	0.029	0.021	0.050	0.051	0.039	0.203	0.185	0.067	0.067	0.027
MaxD^f^ M–Fe^b^	–0.164^**2**^	–0.119^**3**^	–0.130^**3**^	–0.069^**1**^	–0.103^**1**^	0.049^**4**^	–0.060^**1**^	0.084^**4**^	0.138^**5**^	0.140^**5**^	0.122^**5**^	0.240^**5**^	0.273^**5**^	0.176^**5**^	0.116^**4**^	0.059^**4**^
MD M–R^c^	–0.013	–0.005	–0.008	0.017	0.013	0.009	0.015	0.029	0.040	0.031	0.037	0.094	0.071	0.033	0.044	0.019
MAD M–R^c^	0.042	0.035	0.034	0.025	0.030	0.021	0.024	0.033	0.043	0.037	0.039	0.097	0.076	0.039	0.049	0.031
MaxD M–R^c^	0.134^**9**^	0.137^**9**^	0.126^**9**^	0.135^**9**^	0.165^**9**^	0.120^**9**^	0.116^**9**^	0.125^**9**^	0.154^**5**^	0.137^**5**^	0.158^**5**^	0.250^**9**^	0.229^**9**^	0.165^**5**^	0.216^**9**^	0.155^**9**^
FeCSD5																
RMSD^a^	0.019	0.019	0.020	0.020	0.025	0.021	0.024	0.023	0.027	0.026	0.029	0.080	0.054	0.028	0.036	0.036
MD Fe–Fe	–0.021	–0.019	–0.028	0.002	0.008	–0.016	–0.043	–0.016	–0.015	–0.050	–0.034	–0.025	–0.037	–0.037	–0.055	–0.078
MAD Fe–Fe	0.030	0.027	0.028	0.023	0.034	0.018	0.043	0.021	0.022	0.050	0.036	0.039	0.044	0.037	0.055	0.078
MaxD^f^ Fe–Fe	–0.093^**D2**^	–0.055^D2^	–0.086^D2^	–0.042^D2^	0.047^D4^	–0.041^D3^	–0.098^D2^	–0.067^D2^	–0.061^D2^	–0.092^D2^	–0.073^D2^	–0.088^D2^	–0.073^D2^	–0.061^D2^	–0.088^D2^	–0.123^D2^
MD Fe–R^d^	–0.010	–0.010	–0.009	0.008	0.020	–0.008	–0.014	0.007	0.013	–0.020	0.003	0.069	0.036	–0.001	–0.001	–0.028
MAD Fe–R^d^	0.016	0.013	0.013	0.017	0.022	0.015	0.015	0.017	0.021	0.021	0.020	0.079	0.050	0.019	0.021	0.028
MaxD^f^ Fe–R^d^	–0.043^**D2**^	–0.038^**D2**^	–0.037^**D2**^	–0.031^**D2**^	0.050^**D1**^	–0.032^**D5**^	–0.038^**D2**^	0.031^**D2**^	0.042^**D2**^	–0.043^**D3**^	–0.037^**D2**^	0.125^**D4**^	0.082^**D2**^	–0.038^**D3**^	–0.044^**D3**^	–0.053^**D3**^
FeMoco																
RMSD^a^	0.088	0.079	0.079	0.046	0.048	0.040	0.042	0.050	0.071	0.068	0.076	0.241	0.212	0.085	0.107	0.062
MD M–Fe^b^	–0.072	–0.067	–0.076	–0.022	–0.010	–0.015	–0.018	0.024	0.058	0.048	0.059	0.262	0.232	0.081	0.096	0.028
MAD M–Fe^b^	0.072	0.067	0.076	0.024	0.024	0.017	0.020	0.026	0.058	0.048	0.059	0.262	0.232	0.081	0.096	0.028
MaxD M–Fe^b^	–0.114	–0.110	–0.118	–0.045	0.062	–0.044	–0.059	0.048	0.088	0.083	0.099	0.401	0.375	0.143	0.165	0.069
MD M–Re	–0.046	–0.042	–0.038	–0.012	–0.002	–0.007	–0.003	0.016	0.032	0.025	0.037	0.124	0.093	0.037	0.049	0.015
MAD M–R^e^	0.046	0.042	0.038	0.017	0.015	0.014	0.014	0.022	0.036	0.031	0.038	0.128	0.099	0.044	0.054	0.028
MaxD M–R^e^	–0.099	–0.093	–0.082	–0.047	–0.054	–0.040	–0.035	0.056	0.096	0.087	0.087	0.383	0.337	0.128	0.175	0.085

a Root-mean-square deviations (RMSD) of the diamond core of the dimers (or the metal and bridging atoms in the case of **9** and the FeCSD5 complexes) and the [MoFe_7_S_9_C] core of FeMoco.

b M = [Mo, Fe].

c M = [Mo, Fe] and R = [S, C, O].

d R = [S, C].

e M = [Mo, Fe] and R = [S, C]

f The label above the values in the MaxDev rows is the label of the complex that has the largest deviation for the given functional (FeMoD11 or FeCSD5).

Our focus in this section has been to compare structural parameters for spin-coupled iron–sulfur systems related to FeMoco (with less weight given to the smaller FeCSD5 test set). Based on these results, the functionals that give the best error statistics for FeMoD11 as well as for FeMoco itself based on mean absolute deviations of M–M distances are r^2^SCAN, TPSSh, B97-D3, B3LYP*, and ωB97X-D3BJ. We note that a comparison based on max M–M deviations as well as metal–ligand distance deviations are also in favor of these functionals. The ωB97X-D3BJ functional, however, shows such poor performance for the closed-shell complexes in FeCSD5 (MAD of 0.078 Å) that its use cannot be fully recommended. As ωB97X-D3BJ is one of only two functionals tested that exhibits the correct long-range behavior (100% self-interaction free in the long-range) and the relatively low errors for FeMoD11 and FeMoco, exploration of a modified form of ωB97X-D3BJ or other range-separated hybrids may be worthy of further future investigations.

Among the functionals r^2^SCAN, TPSSh, B97-D3, and B3LYP*, we hesitate to further distinguish between them at this stage, though it is noteworthy that r^2^SCAN is the best performing functional for all three test sets in Table 4. The r^2^SCAN functional^97^ is a revised version of the original SCAN functional,^145^ a meta-GGA functional designed to satisfy more exact Kohn–Sham DFT constraints than other functionals. The balanced performance of the functional seen in our comparison (and the lack of expensive exact exchange) and in previous comparisons of both main group and transition-metal test sets^97,146,147^ suggests it as a suitable functional for treating iron–sulfur chemistry and perhaps a balanced description of both transition metal and main group chemistry in general. It seems especially suitable for large metal clusters like FeMoco, where evaluating exact exchange becomes an expensive component of the calculation.

Finally, we note that the M–M distance in FeMoD11 and FeCSD5 may not only be sensitive to the local electronic structure but also to crystal packing effects that may depend both on complex total charges and bulkiness of the ligands. The magnitude of such environmental effects for these molecular crystals will be assessed in future work. Clearly, however, our results strongly imply that the molecular structure of spin-coupled iron–sulfur complexes and clusters favors specific functionals that incorporate either zero or a small amount of exact exchange (0–15%) with considerably worse results seen for functionals with >20% exact exchange.

### Correlation between Bridging Metal–Ligand Bond Lengths and Metal–Metal Distance in FeMoD11

As demonstrated in the previous section, the Fe–Fe/Mo–Fe distances in the FeMoD11 test set are clearly highly sensitive to the exact exchange in the functional (although also to the specific exchange and correlation functionals) with a clear trend with increased exact exchange (going from the underestimation of M–Fe distances to overestimation), while no such trend can be found for the closed-shell test set.

**Figure 7:**
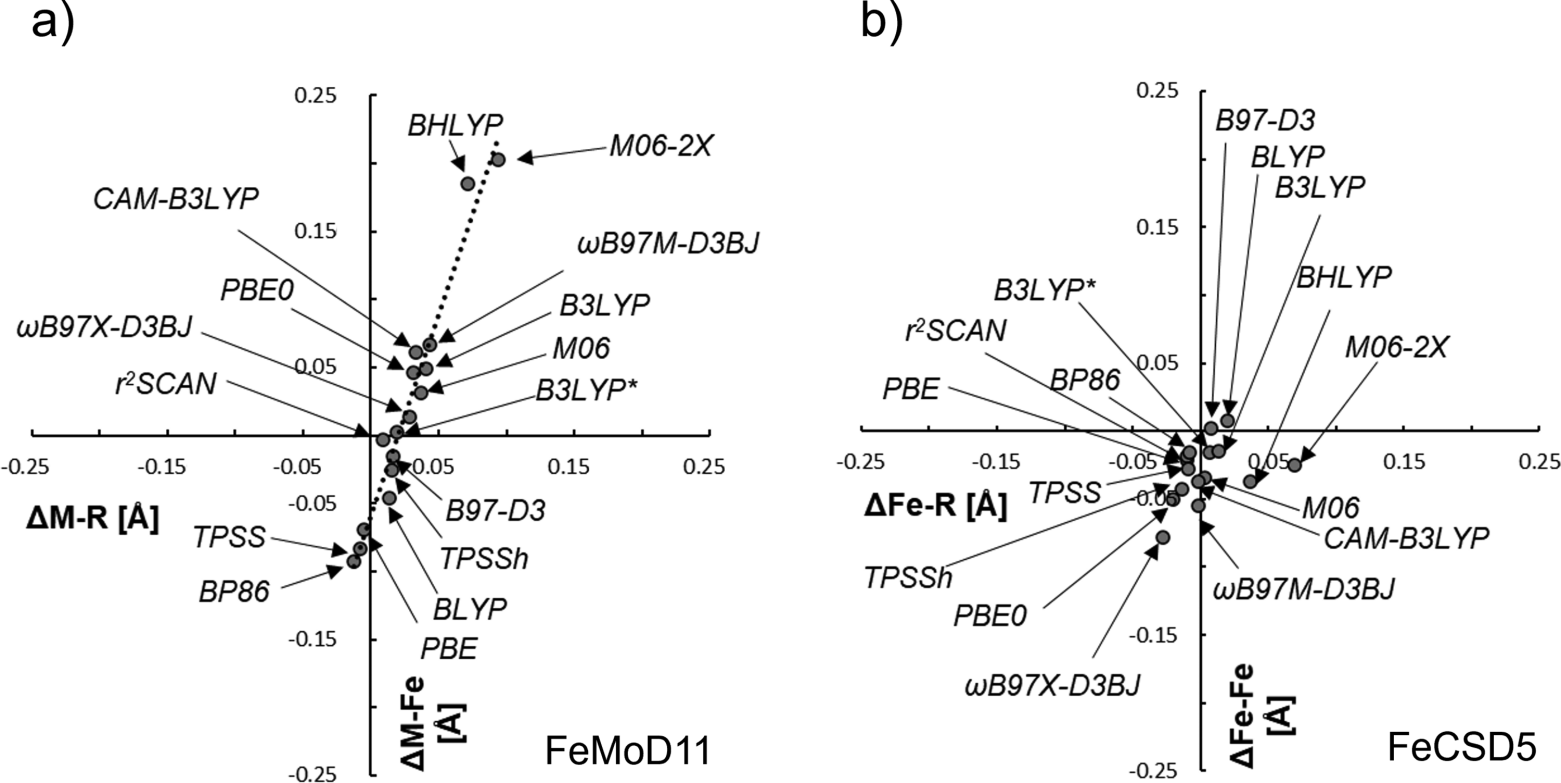
Deviation (Å) in the metal–metal distance, ΔM–Fe, vs the mean deviation in the metal–bridging ligand bond length, ΔM–R, for (a) FeMoD11 and for (b) FeCSD5. For (a), M = Fe, Mo and R = C, O, S, whereas for (b), R = C, S. Linear fit parameters for (a) are *y* = 2.955*x* – 0.0585 with *R*^2^ = 0.958.

The reason for this different behavior between spin-coupled and closed-shell compounds might be rationalized by recognizing the role that the bridging ligand is known to play in the interactions between open-shell metal ions (superexchange and metal–ligand spin polarization) in exchange-coupled dimers.^59^Figure 7 shows the correlation between the deviation of the Fe–Fe/Mo–Fe distance and the deviation for the bridging M–R distances (where R is the bridging ligand atom) for different functionals. The figure is grouped into the spin-coupled M–Fe dimers of FeMoD11 (Figure 7a) and the closed-shell Fe–Fe dimers of FeCSD5 (Figure 7b).

There is an obvious correlation seen for the spin-coupled Fe–Fe and Mo–Fe dimers, suggesting that the errors in Fe–R/Mo–R distances are linked to the errors for the Fe–Fe/Mo–Fe distance. The only exception to this trend is for complex **9** that does not feature a diamond core but instead features three bridging hydroxo groups (see Figure S2 in the SI). Complex **9** is furthermore the only mixed-valence delocalized *S* = 9/2 complex, featuring ferromagnetic coupling (due to double exchange) instead of the antiferromagnetic coupling (typically due to superexchange), and would thus be expected to depend more on direct d-orbital overlap between Fe ions rather than via the ligand-based superexchange mechanism. In sharp contrast, the data for the closed-shell complexes in Figure 7b show no visible trend.

**Figure 8:**
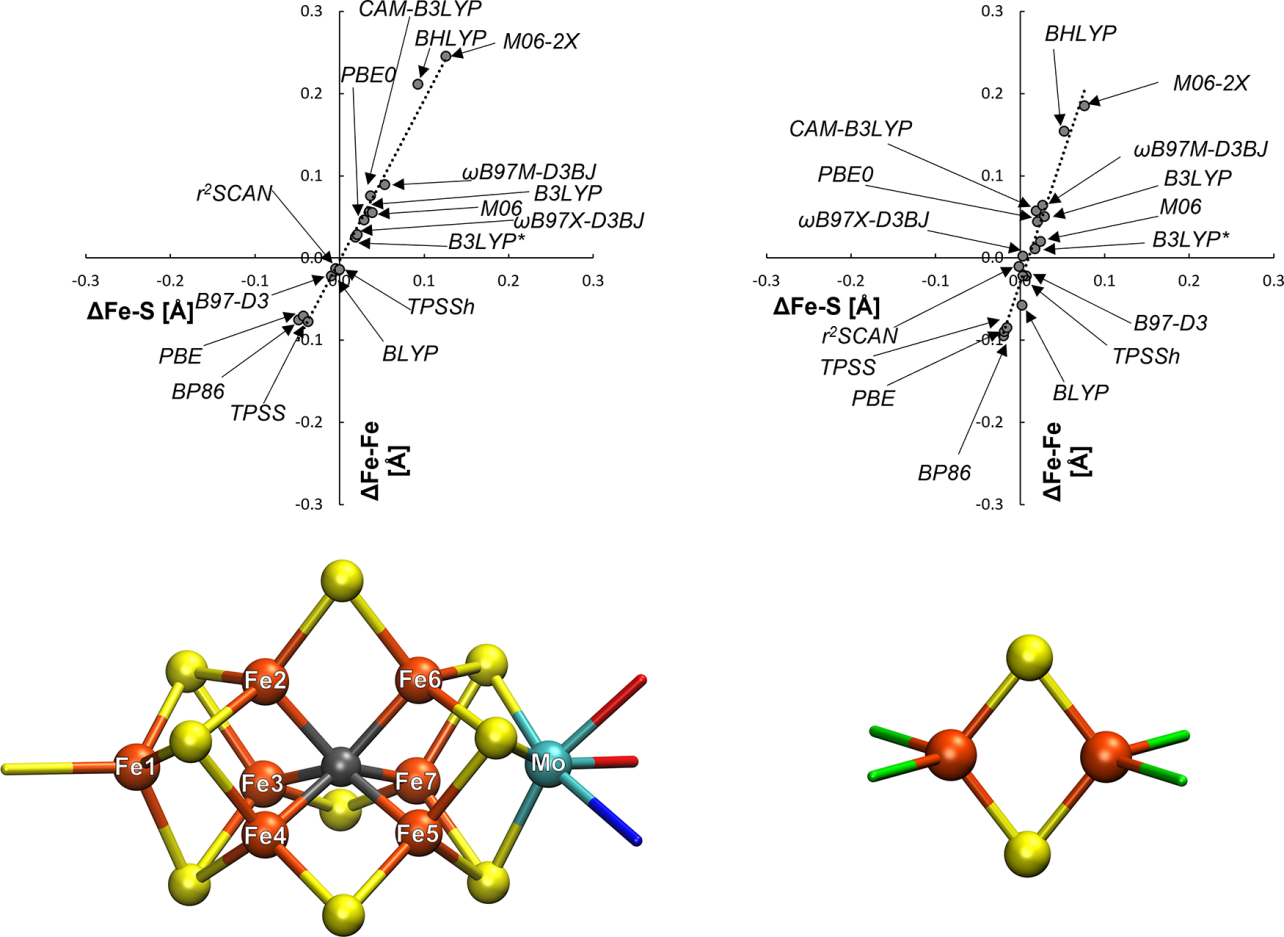
Mean deviation in the Fe–Fe distance (ΔFe–Fe) vs mean Fe–S distance (ΔFe–S) of optimized structures in comparison to the X-ray structures (for FeMoco, PDB: 3U7Q, and for **7**, CSD: EAPFTM01) with the functionals tested. Linear fit parameters *y* = 1.954*x* – 0.003, *R*^2^ = 0.977 (FeMoco) and *y* = 3.085*x* – 0.0301, *R*^2^ = 0.9509 (for complex **7**). FeMoco data come from a 244 QM atom QM/MM model (see the Computational Details section).

Additionally, a highly similar correlation between bridging Fe–S bond length mean errors and Fe–Fe distance mean errors can be seen for FeMoco (Figure 8, left), and this can be compared to the errors for the simplest iron–sulfur dimer in the FeMoD11 test set, complex **7** (Figure 8 right), an Fe(III)–Fe(III) [Fe_2_S_2_Cl_4_]^2–^ complex.

**Figure 9:**
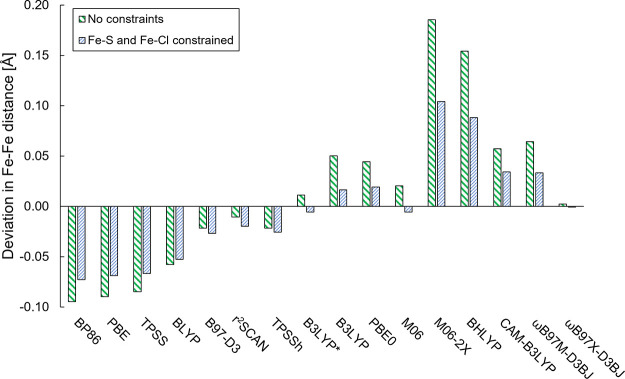
Effect of constraining Fe–S/Fe–Cl distances at the X-ray distances, *r*(Fe–S) = 2.201 and 2.198 Å, whereas *r*(Fe–Cl) = 2.244 and 2.256 Å) on the Fe–Fe distance for [Fe_2_S_2_Cl_4_]^2–^ complex **7** with different functionals.

In our opinion, these correlations arise due to one of two possibilities. The first one is that the spin-coupled electronic structure in the FeMoD11 test set is the reason for these trends (implicating bridging ligand-based superexchange). The second is the correlation being related to the specific geometry of the diamond core of the dimers in FeMoD11, i.e., the bridging M–L distances enforcing a specific Fe–Fe distance (a more direct causal relationship). To clarify this, we carried out constrained geometry optimizations for complex **7** (Figure 8) as a representative of the FeMoD11 test set. By constraining the bridging Fe–S bonds as well as the terminal Fe–Cl bonds to the X-ray structure values (*r*(Fe–S) ≈ 2.20 Å, whereas *r*(Fe–Cl) ≈ 2.25 Å) and optimizing the geometry, we obtain the plot in Figure 9 that shows the *r*(Fe–Fe) distance deviations vs functional for both unconstrained and constrained optimizations. The data for unconstrained and constrained optimizations look overall highly similar, with functionals like BP86, PBE, and TPSS underestimating the Fe–Fe distances by a similar amount whether the Fe–S/Fe–Cl bonds are constrained or not. Meanwhile, the hybrid functionals like M06-2X and BHLYP give strongly overestimated distances even when the Fe–S/Fe–Cl bonds are constrained. Overall, these results suggest that the reason for the trends in Fe–Fe distances of the FeMoD11 test set and the correlation with bridging ligand bond lengths cannot primarily be rooted in a geometric effect of the diamond core (otherwise, the Fe–Fe distance would be predicted to be the same for all functionals when the Fe−S bond is constrained) but instead must arise due to some hidden variables, likely the underlying electronic structure. We note though that the plot also reveals more complex behavior for some of the functionals (the hybrid functionals in particular), where constraining the Fe–S/Fe–Cl bonds to the X-ray distance leads to much smaller Fe–Fe distance deviations than without constraints. There is hence also a geometric effect present involving the diamond core in the calculations, in addition to the electronic structure effect that appears to be responsible for the main method dependency of the results.

The electronic structure effect that should be at play here can be rationalized via a superexchange mechanism that is typically dominant in spin-coupled Fe–S dimers,^59,117^ where spin centers interact via spin polarization of the bridging ligand orbitals. The superexchange interaction arises due to the covalency of the metal–ligand bonds, which are known to be quite dependent on the exact exchange in the functional, which in turn should affect metal–ligand distances. More covalent metal–ligand bonds would thus be expected to give a stronger superexchange interaction, which should bring the metal ions closer to each other. This is particularly noticeable for the TPSS and BP86 data in Figures 7a and 8, where the shortest Fe–R ligand bonds are present and in turn give the shortest Fe–Fe distances.

### Correlation between Fe–S Bond Covalency and Fe–Fe Distance

The correlations in the previous section imply that the treatment of the bridging ligand–metal bond (Fe–S bond in most of the complexes) is important for an overall accurate treatment of the metal–metal interaction. To gain more insight into this correlation, we have analyzed the electronic structure of complex **7** in detail. This structurally simple 2Fe(III) complex features an antiferromagnetically coupled *S* = 0 ground state (described by an *M*_S_ = 0 determinant with BS-DFT), and the deviations found for different functionals correlate overall quite well for the deviations for the whole test set and even to FeMoco (compare Figures 7 and 8), with BP86 and TPSS underestimating the Fe–Fe distance by 0.08–0.09 Å, r^2^SCAN (Δ = −0.01 Å), TPSSh (Δ = −0.02 Å), B3LYP* (Δ = +0.015 Å) and ωB97X-D3BJ (Δ = +0.002 Å) showing smaller deviations and other functionals overestimating the Fe–Fe distance from +0.02 Å (M06) to +0.18 Å (M06-2X). The Fe–Fe distance is highly sensitive to the total spin and thus the nature of the coupling, and we note that if the ferromagnetic *M*_S_ = 5 state is calculated instead, the distance increases to 2.95 Å at the TPSSh level, in sharp contrast to the 2.70 Å using the antiferromagnetic *M*_S_ = 0 broken-symmetry state. This both demonstrates that the use of a ferromagnetic state (featuring less spin contamination but the wrong spin state) in geometry optimizations is not a useful approach for iron–sulfur systems and also that the Fe–Fe distance trends discussed must be primarily related to the electronic structure and the specific nature of the spin-coupling.

**Figure 10:**
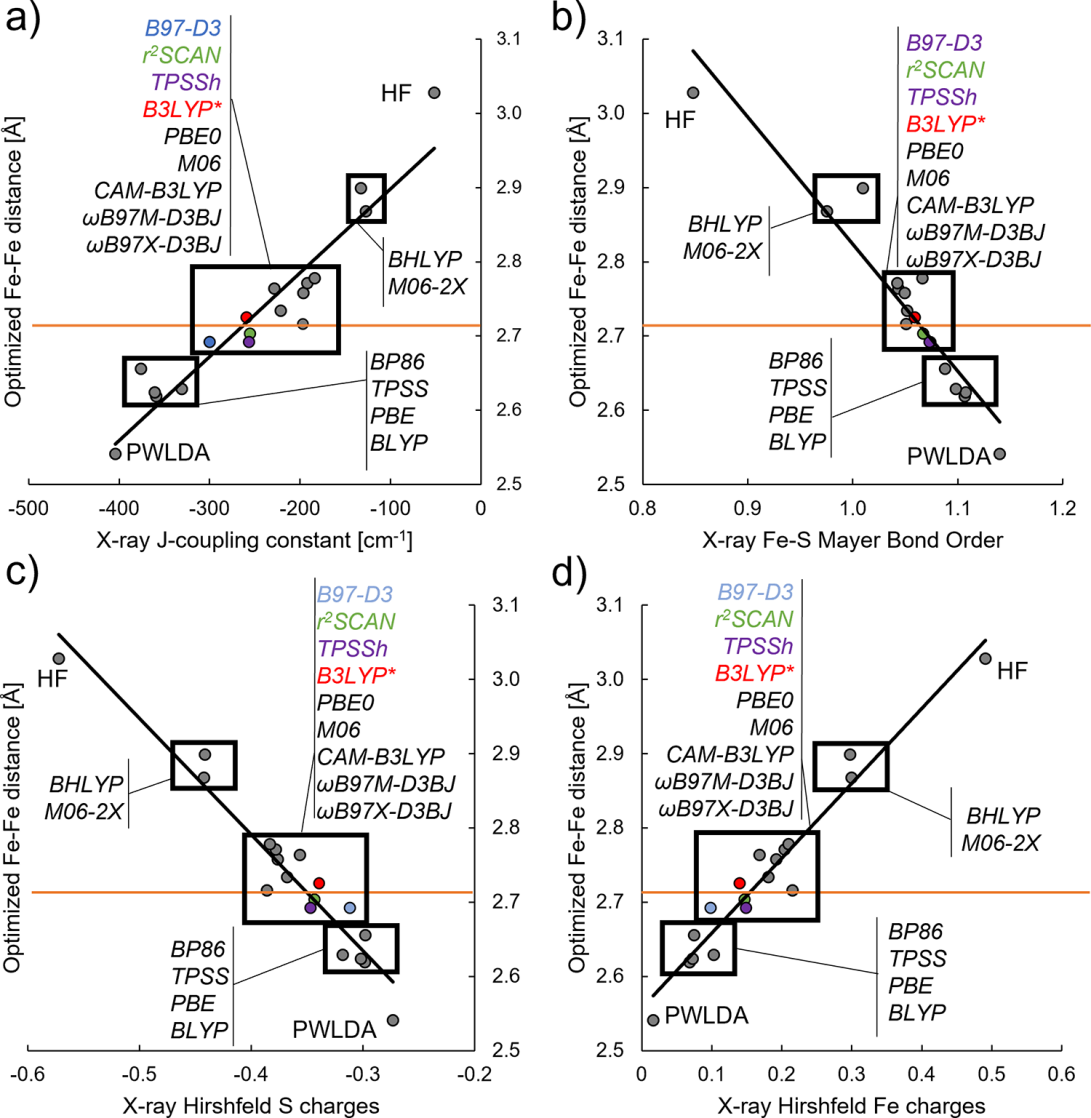
Correlation plots of the optimized Fe–Fe distance (*y*-axis) of **7** vs various parameters evaluated on the X-ray structure (*x*-axis): *J*-coupling, Fe–S Mayer bond order, Hirshfeld S charge, or Hirshfeld Fe charge. (a) *J*-coupling constant evaluated in cm^–1^ according to the Yamaguchi equation,^55,56^ (b) the calculated Fe–S Mayer bond order, (c) average Hirshfeld charge on sulfides, and (d) average Hirshfeld charge on Fe. The red line indicates the X-ray Fe–Fe distance. Certain functionals are color-coded (the gray dot which the orange line crosses is ωB97X-D3BJ). Values are tabulated in Table S1.

The Fe–Fe distance for **7** calculated with different functionals correlates well with the calculated exchange coupling constant *J* (Figure 10a) when calculated via the Yamaguchi equation^55,56^ via single-point energy evaluation on the X-ray structure. As previously mentioned, the Fe–Fe distance also correlates with the average Fe–S distance errors in the [Fe_2_S_2_] core (see Figure 8). Suspecting Fe–S bond covalency to be the underlying cause behind these correlations, we calculated simple electronic structure parameters with an obvious connection to metal–ligand bond covalency: Hirshfeld charges and Mayer bond orders. Importantly, the Hirshfeld charges and Mayer bond order were evaluated on the X-ray geometry of the complex with each functional rather than an optimized structure. Plotting the calculated Hirshfeld S atomic charge against the optimized Fe–Fe distance for each functional (Figure 10c) results (Figure 10d) in an inverse correlation (more negative S-charge, longer Fe–Fe distance) while the Hirshfeld Fe atomic charge gives a regular correlation (more positive Fe charge, longer Fe–Fe distance). An even better correlation is observed when the Fe–S Mayer bond order is plotted against the Fe–Fe distance (Figure 10b). Adding the local density functional, PWLDA, as well as the HF method to the correlation plots in Figure 10 shows that these correlations hold, even for methods that strongly favor delocalization (PWLDA) or localization (HF). These correlations clearly suggest the covalency of the Fe–S bond to be responsible for the functional dependency of the Fe–Fe distance as the S/Fe atomic charge or Fe–S Mayer bond order changes appreciably when evaluated with each functional (or HF) on the same X-ray structure geometry. Different degree of covalency of the bridging Fe–S bond would thus result in different magnitude of the superexchange interaction between Fe ions, leading to a different Fe–Fe distance.

**Figure 11:**
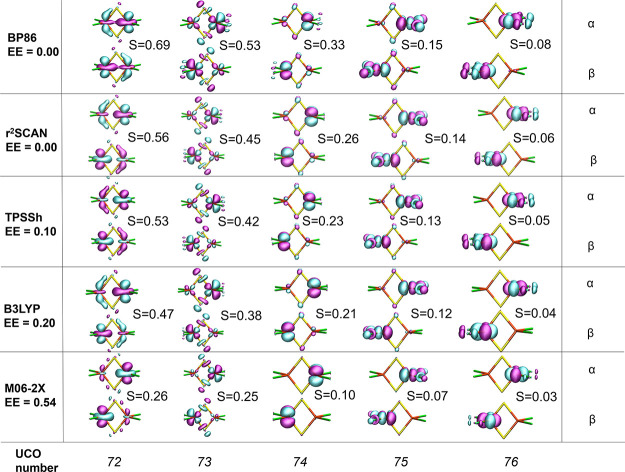
Unrestricted corresponding orbitals (UCOs) of **7**. The UCOs are derived from single-point calculations with each respective functional on the X-ray crystal structure geometry. *S* indicates the overlap between the α and β orbitals. A contour value of 0.05 was used for the orbital isosurfaces.

A different insight into the superexchange mechanism can be obtained via the corresponding orbital transformation of the broken-symmetry calculation. This offers a convenient valence-bond like the description of the broken-symmetry determinant and leads to a clear distinction of the orbitals of the system into doubly-occupied α–β orbital pairs (overlap close to unity), nonorthogonal spin-coupled orbital pairs (overlap <1 and >0), and unpaired uncoupled α orbitals (overlap close to zero). Figure 11 shows isosurfaces of selected corresponding orbitals (having overlap between 0 and 1) of **7**, calculated on the X-ray geometry with five different density functionals. The overlap values for all functionals in this study are present in Table 5. These magnetic orbitals correspond well to the Fe 3d-orbitals of the system, while clearly showing the contribution of bridging sulfide character that is responsible for the superexchange interaction. While the orbitals remain qualitative similar for all five functionals, there is a considerable difference in the overlap itself as well as the bridging sulfide contribution to all unrestricted corresponding orbitals (UCOs); the differing amount of exact exchange likely behind the largest differences. For UCO pairs 74–76, the overlap is fairly small (these orbitals would contribute the least to the spin coupling) and always larger for the nonhybrid functionals compared to the hybrid functionals. UCO pair 72–73 shows the largest differences in terms of overlap and bridging sulfide character and clearly indicates the importance of superexchange in the spin coupling. Intriguingly, the BP86 functional reveals the UCO 72 pairs as having an unusually large overlap (*S* = 0.69) and the shapes of the orbital isosurfaces suggest even some direct overlap of the d-orbital part of the two Fe ions. This would indicate a possible direct-exchange interaction or perhaps even partial metal–metal bonding present in the BP86 calculation, and it is easy to imagine how maximizing this orbital overlap in UCO pair 72 might then lead to a considerable Fe–Fe contraction in this complex. In fact, optimizing the structure at the BP86 level is found to give a Fe–Fe distance shortening of −0.09 Å compared to the X-ray structure. This shortening leads to a change in the overlap of UCO pair 72 from *S* = 0.69 to 0.74. The changes in the overlap upon structure relaxation for the other functionals can be found in Table S2 in the SI, while Figure S5 shows how the individual UCO orbital overlaps for different functionals correlate with Fe–Fe distance.

**Table 5:** Overlaps of the Five Unrestricted Corresponding Orbital Pairs with 3d Character (See Figure 11), Evaluated on the X-ray Geometry with Different Functionals

	overlap				
functional	UCO 72	UCO 73	UCO 74	UCO 75	UCO 76
PWLDA	0.73	0.58	0.39	0.18	0.09
BP86	0.69	0.54	0.35	0.17	0.08
PBE	0.69	0.54	0.35	0.16	0.07
TPSS	0.66	0.52	0.32	0.16	0.06
BLYP	0.71	0.56	0.38	0.16	0.07
B97-D3	0.61	0.48	0.31	0.15	0.07
r^2^SCAN	0.56	0.45	0.26	0.14	0.06
TPSSh	0.53	0.42	0.24	0.14	0.05
B3LYP*	0.52	0.41	0.24	0.14	0.05
B3LYP	0.47	0.38	0.21	0.12	0.04
PBE0	0.41	0.34	0.18	0.11	0.04
M06	0.42	0.34	0.20	0.12	0.04
M06-2X	0.26	0.25	0.11	0.08	0.03
BHLYP	0.27	0.25	0.11	0.08	0.02
CAM-B3LYP	0.40	0.34	0.18	0.11	0.03
ωB97M-D3BJ	0.38	0.32	0.16	0.10	0.03
ωB97X-D3BJ	0.41	0.33	0.17	0.10	0.03
HF	0.12	0.11	0.05	0.03	0.01

The rather unusually strong overlap for UCO pair 72 for complex **7** and the general underestimation of Fe–Fe distances seen for **7** and the overall FeMoD11 test set statistics hence indicate that the covalency or delocalization is overestimated in some of the nonhybrid functionals (likely due to the well-known self-interaction and delocalization error that plagues these functionals), leading to an exaggerated Fe–Fe interaction. The problem is reversed in the case of a functional like M06-2X, where the reduced Fe–S covalency leads to reduced favorable superexchange interactions and hence longer Fe–Fe distances. We hypothesize based on these results that the reason for the more favorable geometric statistics of r^2^SCAN, TPSSh, B97-D3, and B3LYP* for the FeMoD11 test set as well as FeMoco is thus likely to reside in a more accurate treatment of Fe–S bond covalency in these spin-coupled Fe–S systems. As Table 5 shows, these four functionals have UCO overlaps relatively close together.

## Conclusions

The Fe–Fe and Mo–Fe distances of spin-coupled dimeric systems studied in this work are revealed to be highly sensitive to the density functional employed, specifically to the amount of exact exchange present in the functional definition, and also to the underlying GGA or meta-GGA exchange–correlation components. The results reveal that the common nonhybrid functionals (such as BP86, PBE, and TPSS) systematically underestimate Fe–Fe/Mo–Fe distances, while the common hybrid functionals with >20% exact exchange instead overestimate these distances. Four functionals, r^2^SCAN, B97-D3, TPSSh, and B3LYP*, with 0–15% exact exchange are found to give the lowest errors for the spin-coupled test set (FeMoD11). r^2^SCAN gives the lowest errors overall for the FeMoD11 test set, FeMoco itself, and a closed-shell Fe–Fe test set for comparison.

Geometric effects in BS-DFT calculations of spin-coupled systems are not discussed much in the literature, probably as spin coupling is typically thought to be a rather weak interaction. Even more generally, molecular geometries are often assumed not to be very sensitive to the DFT method and a common practice is to use a lower level of theory (e.g., nonhybrid functionals) to optimize geometries while a higher level of theory (e.g., hybrid functionals or wavefunction theory (WFT) methods) used to calculate more accurate reaction energies or spectroscopic properties on the low-level geometry. This common practice (while undoubtedly successful for many systems) is unlikely to be a useful strategy for spin-coupled iron–sulfur complexes, as these systems clearly exhibit a strong functional dependence of the calculated electronic structure that further translates into a strong functional dependence of the molecular structure. It is, e.g., not clear what a single-point energy calculation of an iron–sulfur compound with the M06-2X functional (predicting strong overestimation of Fe–Fe distance) on a BP86-calculated geometry (predicting fairly strong underestimation of the Fe–Fe distance), as an extreme example, would really describe, seeing as the two functionals predict very different electronic structures and geometries, rendering the energy surface ill-defined.

The effects seen for these spin-coupled systems are large in magnitude, which is very likely due to the strong connection between covalency and superexchange and due to the more flexible metal–ligand bond involving a 3p element (S) than a 2p element (e.g., an oxo bridge). Fe–S covalency as an important metric in DFT calculations of iron–sulfur clusters has been previously discussed by Szilagyi and co-workers.^75,76^

Two caveats regarding our results should be mentioned: (1) We compare DFT-calculated geometries calculated with a polarizable continuum model with X-ray crystal structures. Crystal packing effects have not been considered in the calculations of FeMoD11 and FeCSD5 test sets (though we note that the FeMoco calculations presented include protein environmental effects via QM/MM) and may have non-negligible effects on some of the molecules considered that would slightly affect the error statistics. Based on preliminary data, we expect crystal packing effects to be larger in magnitude for the bulkier complexes while smaller systems such as complex **7** should be less affected. (2) We utilize broken-symmetry determinants in this work that are not eigenfunctions of the total spin operator. This leads to artificial α and β spin densities being present in the calculations of the antiferromagnetically coupled singlet states (a real singlet has no spin density). It is unclear what the effects of not preserving spin symmetry are in BS-DFT geometry optimizations, especially since the total spin operator can only be applied to the noninteracting Kohn–Sham wavefunction. Future work may consider the use of spin projection gradients that have recently been utilized by Guidoni and co-workers for iron–sulfur clusters.^63,70,71^ However, it remains unclear how appropriate these spin projection schemes (that assume the validity of HDVV spin Hamiltonians) are for the more complex covalent and often delocalized electronic structure exhibited by iron–sulfur clusters.

In this study, we have focused on the usefulness of analyzing geometries of spin-coupled iron–sulfur complexes and shown that a density functional that predicts an accurate geometry (as primarily judged by the distance between the spin-coupled Fe–Fe/Mo–Fe ions) describes a specific electronic structure that primarily relates to the covalency of the bridging iron–sulfur bond. In our view, this strongly implies that a functional that predicts accurate geometries for spin-coupled iron–sulfur systems is describing the electronic structure of these systems more accurately than other methods and should in turn be more suitable to describe the full potential energy surface of these systems. However, the molecular structure can also not reveal the full picture of the accuracy of the electronic structure and for the four functionals that emerged from our comparison: r^2^SCAN, TPSSh, B97-D3, and B3LYP*, we might not expect identical trends for reaction energies or other properties, as the functional components are rather different (GGA vs meta-GGA, 0% EE vs 10% EE vs 15% EE, etc.). Nonetheless, an accurate treatment of iron–sulfur bond covalency (that as shown affects the molecular structure) should be a prerequisite for obtaining the right result for the right reason with a quantum chemistry method.

For describing energetics related to the complex mechanism of dinitrogen reduction to ammonia by the FeMoco cluster of nitrogenase, errors associated with redox energies, protonation energies, metal hydride bond formation energies, N_2_ and H_2_ binding energies, and metal–sulfur bond dissociation energies also need to be evaluated. Some recent studies by Dance and Ryde and co-workers have been devoted to the topic of benchmarking properties related to nitrogenase reactions,^148,149^ where density functionals were compared for reactions, structures, and vibrational frequencies involving low-spin, low-valent organometallic compounds with strong-field ligands (primarily CO). We note, however, that FeMoco features high-spin Fe and Mo ions in a weak-field sulfide environment instead and that benchmarking energy errors for high-spin metal ions are likely more relevant than low-spin metal ions.

## Supporting Information Available

Plots of data with MAD instead of MD; plots of structure deviations for each functional or each molecule; and all calculated distances and electronic structure parameters (PDF)
*XYZ* coordinates of all compounds (ZIP)
