# Supplementary material for: Analysis of the Geometric and Electronic Structure of Spin-Coupled Iron–Sulfur Dimers with Broken-Symmetry DFT: Implications for FeMoco
Source: J Chem Theory Comput. 2022 Feb 15;18(3):1437–57. doi: 10.1021/acs.jctc.1c00753 (PMC8908755; doi:10.1021/acs.jctc.1c00753)
Supplement: Supplementary file 1 — ct1c00753_si_001.pdf [file ct1c00753_si_001.pdf]

Supporting Information for:
Analysis of the geometric and electronic structure of spin-coupled iron-sulfur dimers with broken-symmetry DFT: implications for FeMoco

Bardi Benediktsson[a], Ragnar Bjornsson[a,b]*

[a] Science Institute, University of Iceland, Dunhagi 3, 107 Reykjavik, Iceland.

[b] Max Planck Institute for Chemical Energy Conversion, Stiftstrasse 34-36, 45470 Mülheim an der Ruhr, Germany

* E-mail: ragnar.bjornsson@cec.mpg.de

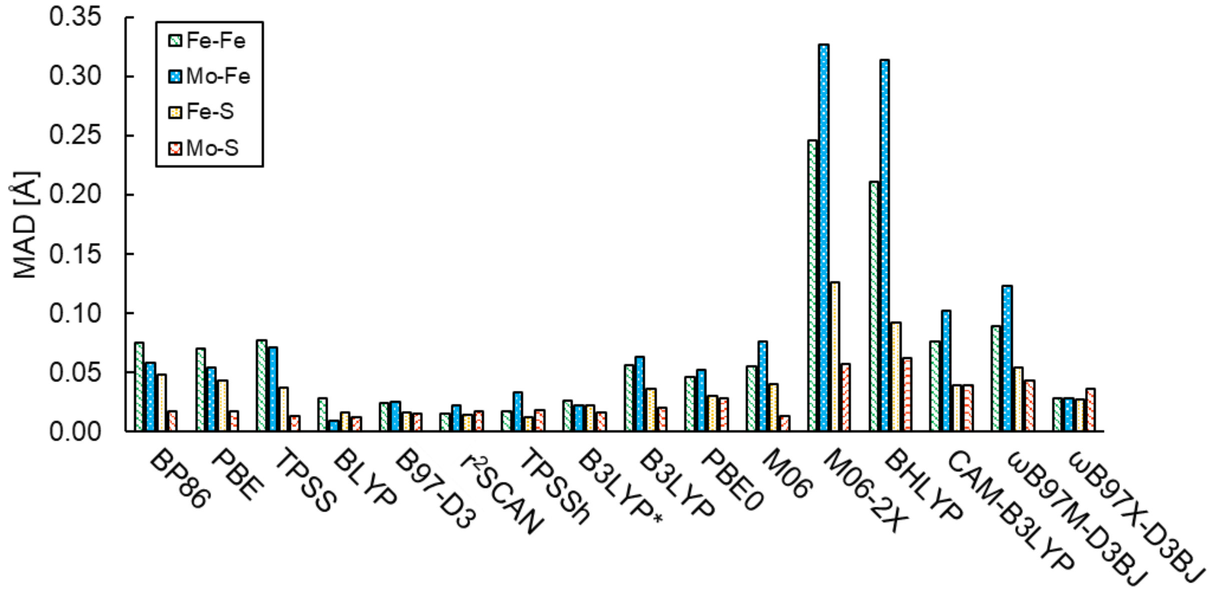

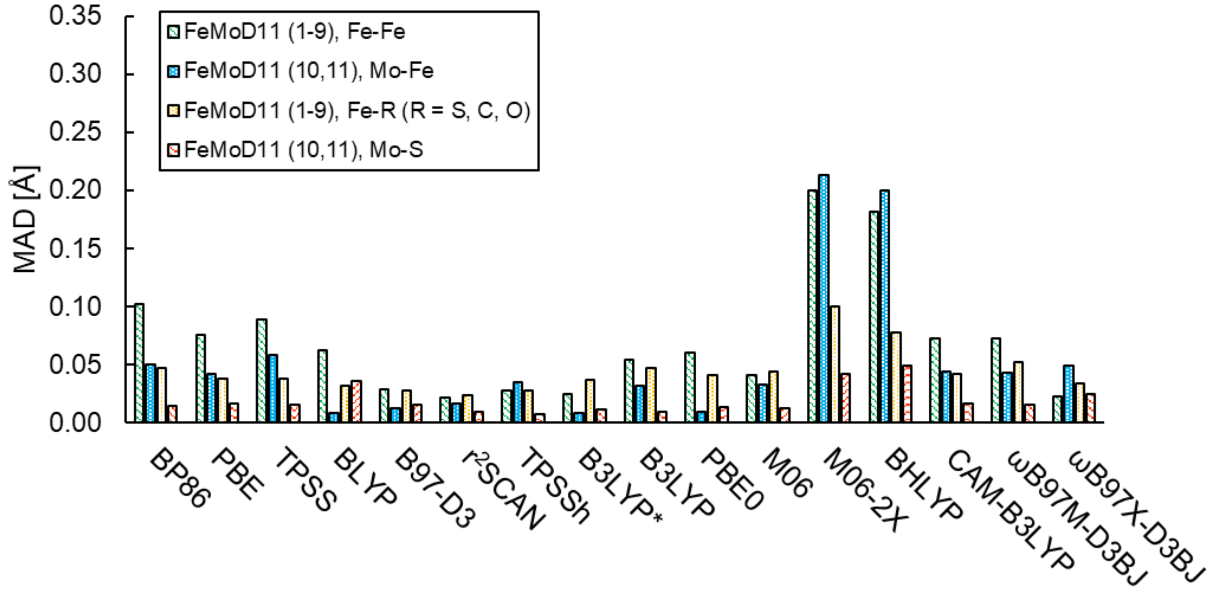

Figure S1: Top: mean absolute deviations (Å) of calculated Fe-Fe, Mo-Fe, Fe-S, and Mo-S distances of FeMoco (244 QM region QM/MM model) for different functionals, with respect to the X-ray crystal structure. Bottom: mean absolute deviations of calculated Fe-Fe, Mo-Fe, Fe-S, and Mo-S distances in the FeMoD11 test set.

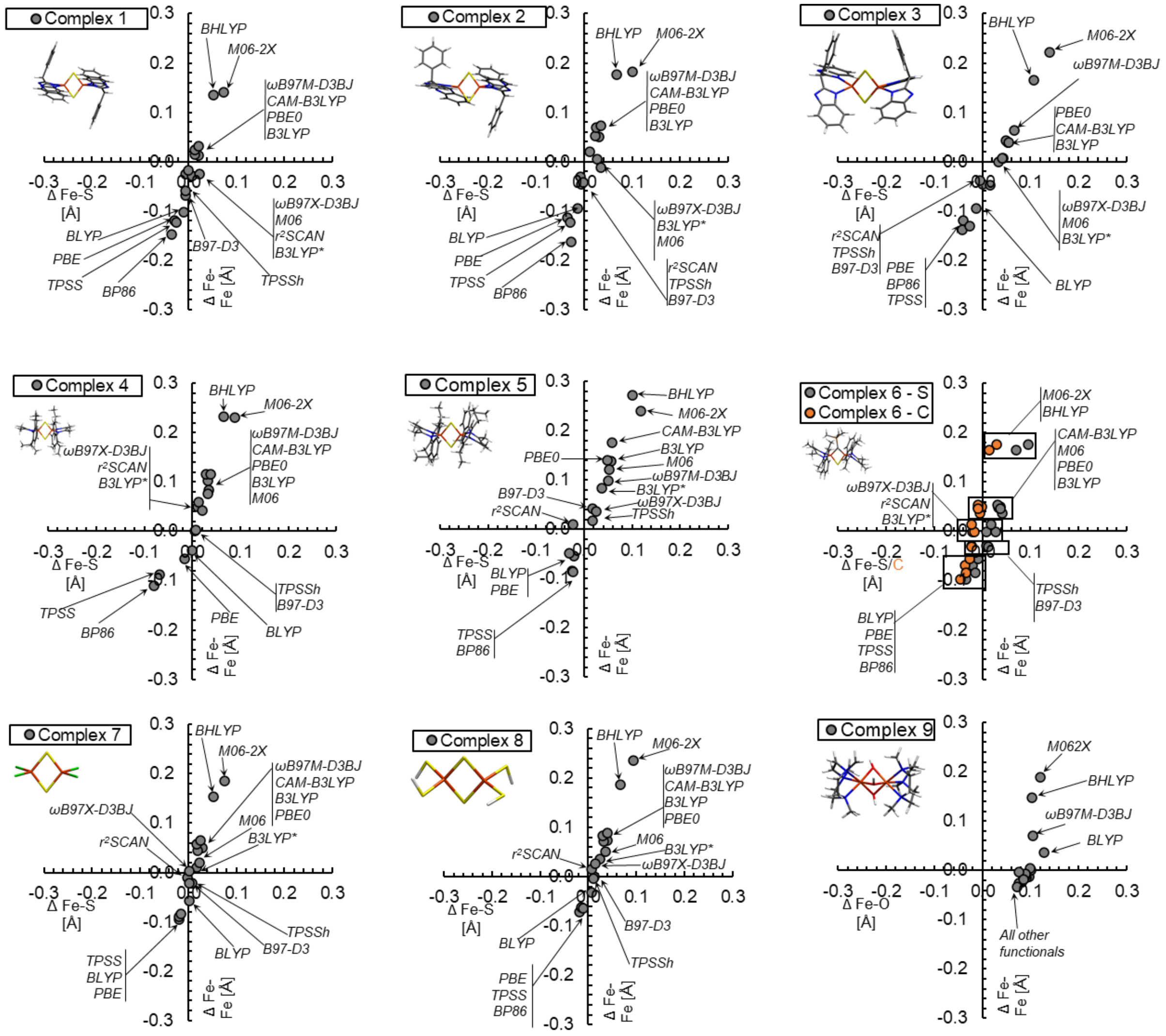

*Figure S2: Deviation of calculated Fe-Fe distance (Å) from the X-ray structure as a function of the mean deviation of the Fe-R bond lengths (Å) for complexes **1**-**9** of FeMoD11 with different functionals (in case of **6**, the grey circles are Fe-C bond lengths).*

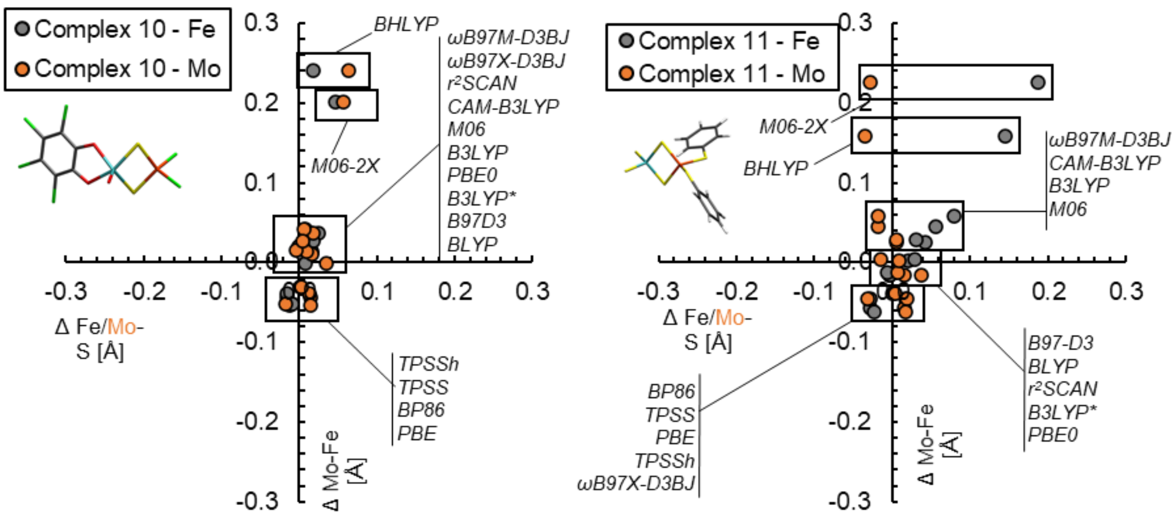

*Figure S3: Deviation of calculated Mo-Fe distance (Å) from the X-ray structure as a function of the mean deviation of the Fe-R bond lengths (Å) for complexes **10** and **11** of FeMoD11 with different functionals (the grey circles are Mo-S bond lengths).*

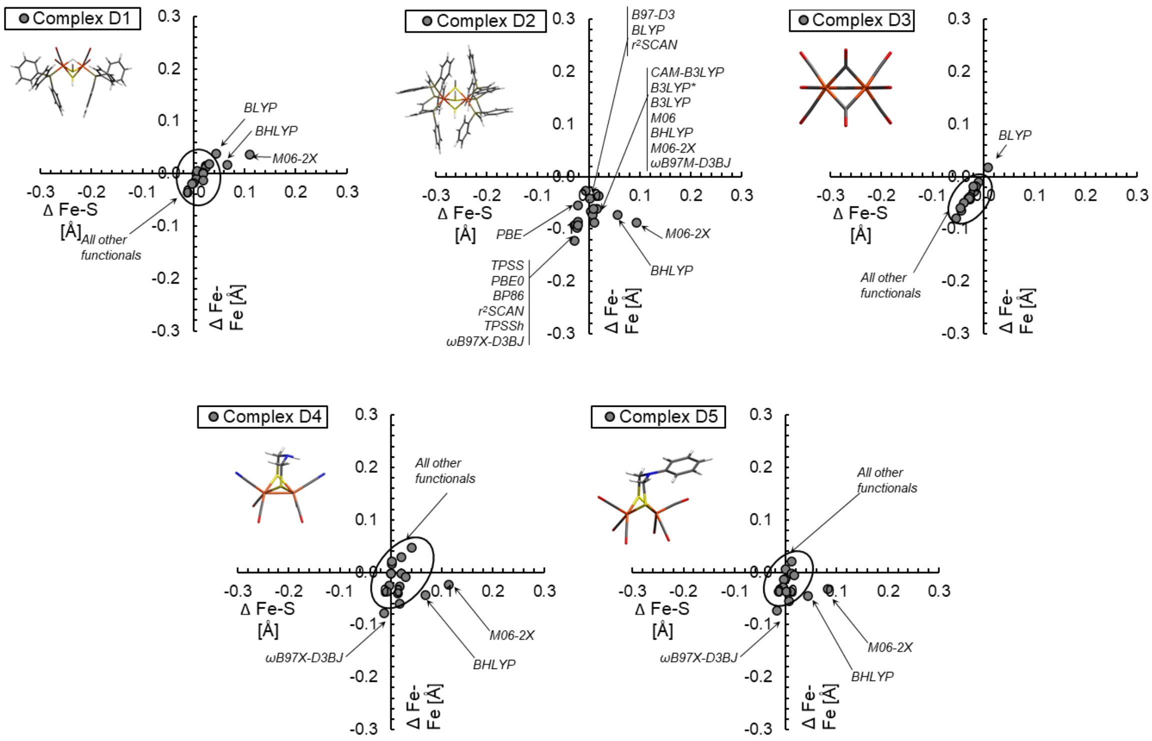

*Figure S4: Deviation of calculated Fe-Fe distance (Å) from the X-ray structure as a function of the mean deviation of the Fe-R bond lengths from the X-ray structure (Å) for complexes **D1**-**D5** of FeCSD5 with different functionals.*

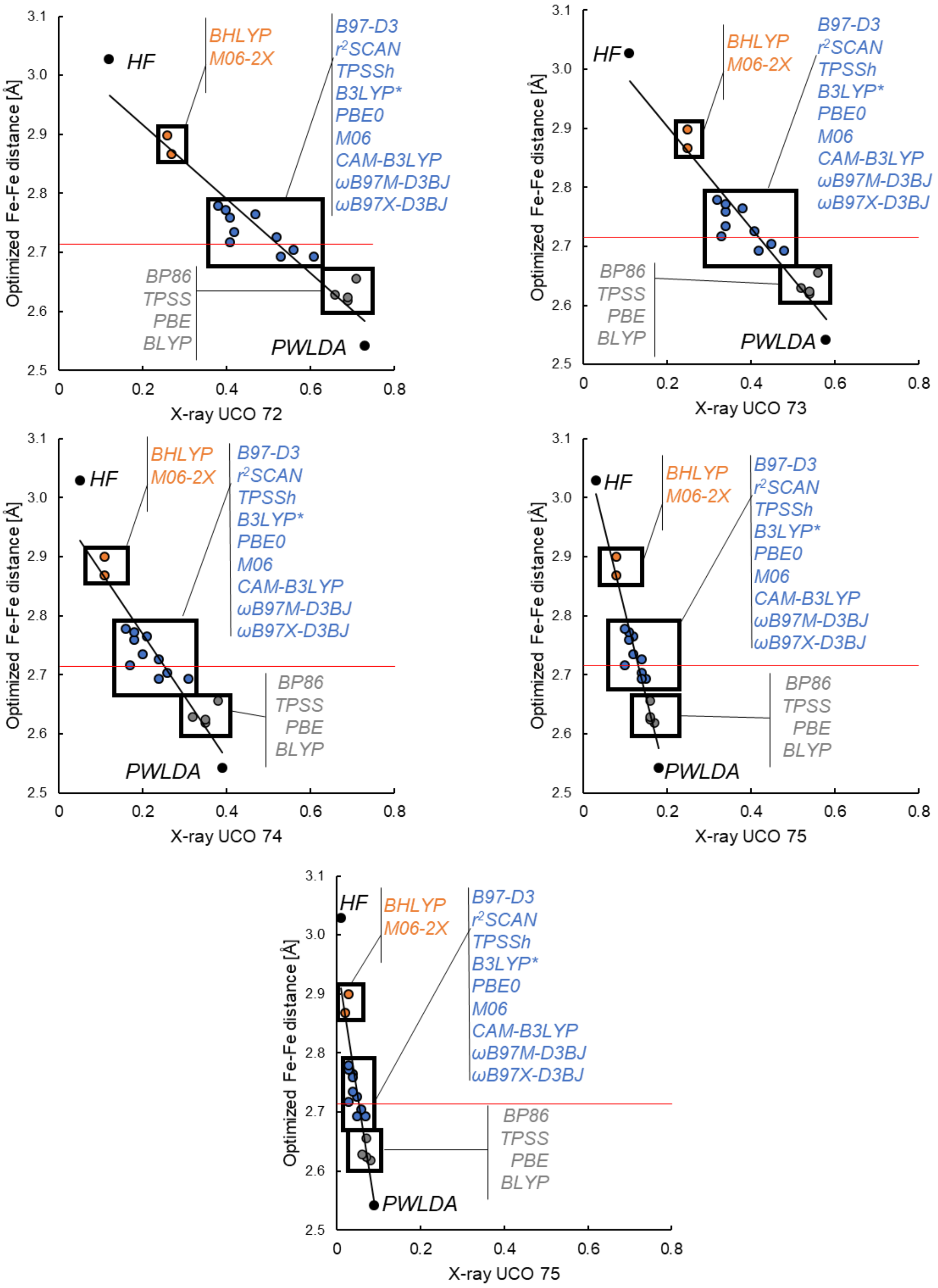

*Figure S5: Optimized Fe-Fe distance (Å) of **7** as a function of different UCO overlaps (see definition of UCO numbers in the main text) from a single point CPCM calculation of the X-ray structure.*

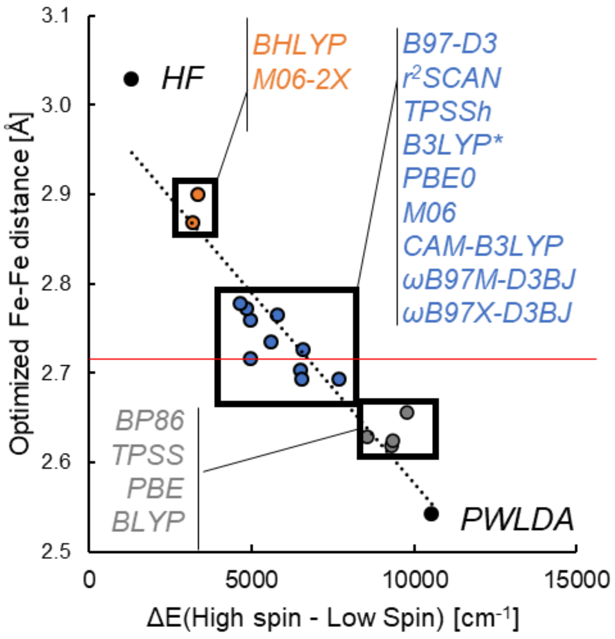

Figure S6: Optimized Fe-Fe distance (Å) of ***7*** *as a function of high-spin/low-spin energy difference from a single point calculation of the X-ray structure.*

*Table S1: Fe-S Mayer bond orders, Hirshfeld charges on Fe, S and Cl, J-coupling constants (cm-1) with different functionals evaluated on the X-ray crystal structure of complex **7**.*

	BP86	PBE	TPSS	BLYP	B97-D3	r²SCAN	TPSSh	B3LYP*	B3LYP	PBE0	M06	M062X	BHLYP	CAM-B3LYP	ωB97M-D3BJ	ωB97X-D3BJ
Fe-S MBO[a]	1.11	1.11	1.10	1.09	1.07	1.07	1.08	1.06	1.04	1.05	1.05	1.01	0.98	1.04	1.07	1.05
Ave. Fe HC[b]	0.07	0.07	0.10	0.07	0.10	0.14	0.15	0.14	0.17	0.19	0.18	0.30	0.30	0.20	0.21	0.22
Ave. S HC[b]	-0.30	-0.30	-0.32	-0.30	-0.31	-0.34	-0.35	-0.34	-0.36	-0.38	-0.37	-0.44	-0.44	-0.38	-0.38	-0.39
Ave. Cl HC[b]	-0.38	-0.38	-0.39	-039	-0.39	-0.40	-0.40	-0.40	-0.41	-0.41	-0.41	-0.43	-0.43	-0.41	-0.41	-0.41
J[c] [cm^{-1}]	-359	-361	-331	-376	-300	-255	-256	-259	-228	-196	-222	-133	-127	-192	-184	-197
dE(H-L)[d] [cm^{-1}]	9313	9347	8547	9764	7715	6537	6541	6610	5805	4976	5618	3343	3197	4862	4643	4986

[a]Mayer Bond Order
[b]Hirshfeld charge
[c]Yamaguchi J-coupling
[d]Difference in energy between the high spin solution and the low spin broken symmetry solution.

*Table S2: The UCO overlaps with different functionals of the the 5 spin-coupled corresponding orbitals (UCOs: see main text) of complex **7** using the optimized geometry instead of the X-ray geometry.*

	Overlap				
Functional	72	73	74	75	76
BP86	0.74	0.57	0.36	0.13	0.07
PBE	0.73	0.56	0.36	0.13	0.07
TPSS	0.70	0.53	0.33	0.13	0.06
BLYP	0.72	0.56	0.39	0.14	0.07
B97-D3	0.61	0.48	0.31	0.15	0.07
r²SCAN	0.56	0.45	0.26	0.14	0.06
TPSSh	0.54	0.42	0.24	0.13	0.05
B3LYP*	0.51	0.41	0.24	0.13	0.05
B3LYP	0.44	0.36	0.20	0.13	0.05
PBE0	0.39	0.33	0.17	0.11	0.04
M06	0.41	0.33	0.19	0.11	0.04
M06-2X	0.22	0.21	0.09	0.08	0.03
BHLYP	0.22	0.22	0.10	0.08	0.03
CAM-B3LYP	0.38	0.32	0.17	0.11	0.04
ωB97M-D3BJ	0.34	0.31	0.15	0.10	0.03
ωB97x-D3BJ	0.39	0.32	0.16	0.10	0.03

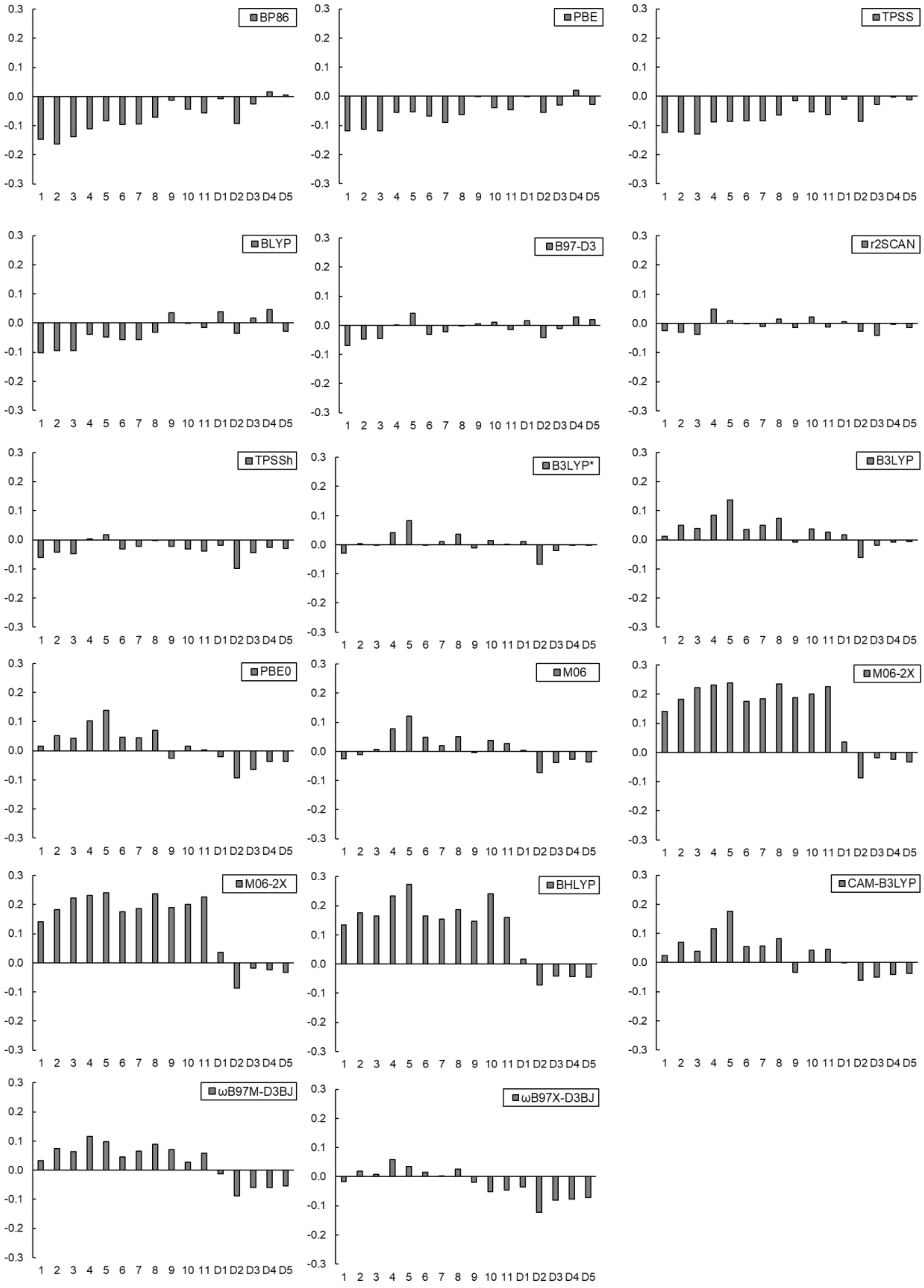

Figure S7: Fe-Fe distance deviations (Å) for all complexes in FeMoD11 and FeCSD5 for each functional tested.

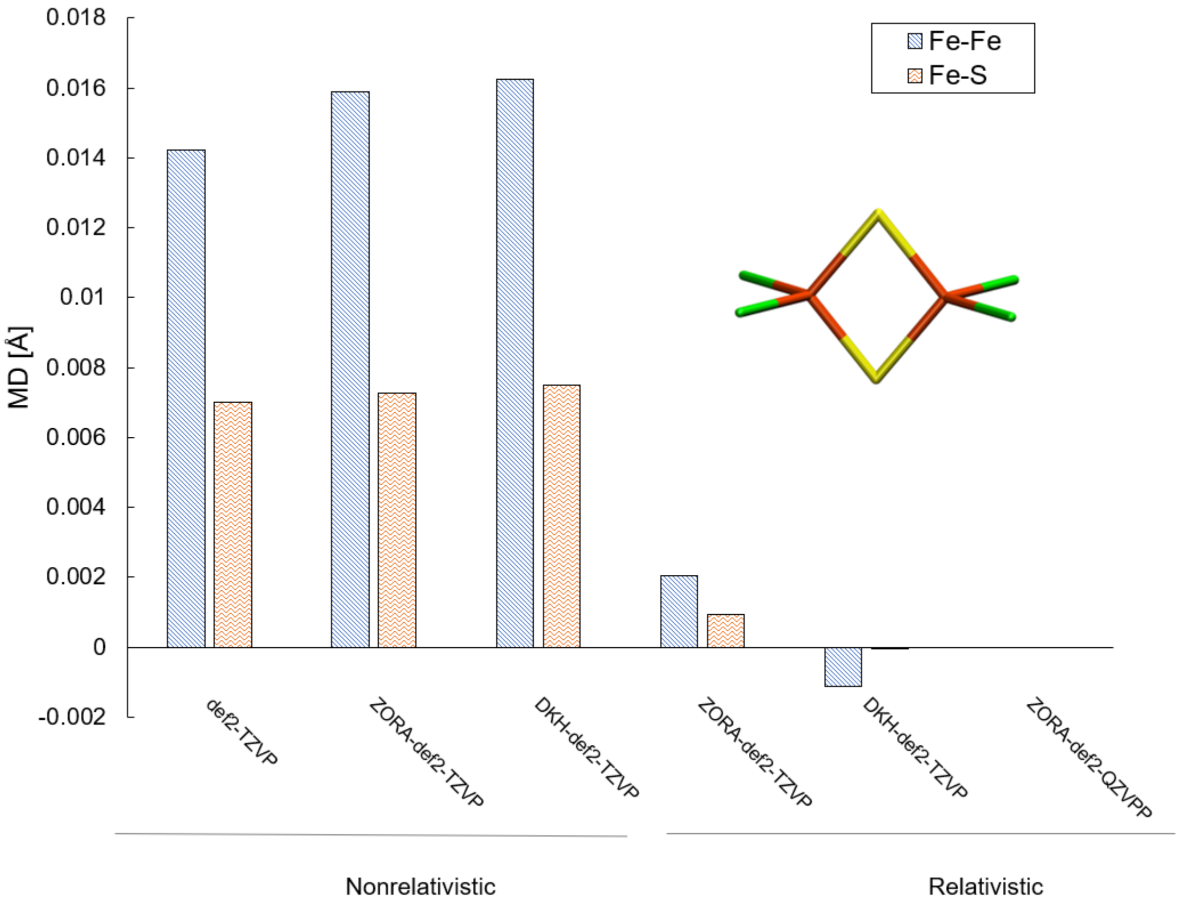

*Figure S8: Mean deviations of Fe-S bond lengths and Fe-Fe distance of complex **7**, $[Fe_2S_2Cl_4]^{2-}$, with and without a scalar relativistic ZORA Hamiltonian or relativistic DKH Hamiltonian with regular Ahlrichs basis set (def2-TZVP) or relativistically reconstracted basis sets for each Hamiltonian (DKH or ZORA). Deviations are relative to the relativistic ZORA-def2-QZVPP reference (r(Fe-Fe) = 2.690 Å and r_{ave}(Fe-S) = 2.202 Å). The TPSSh functional was used.*

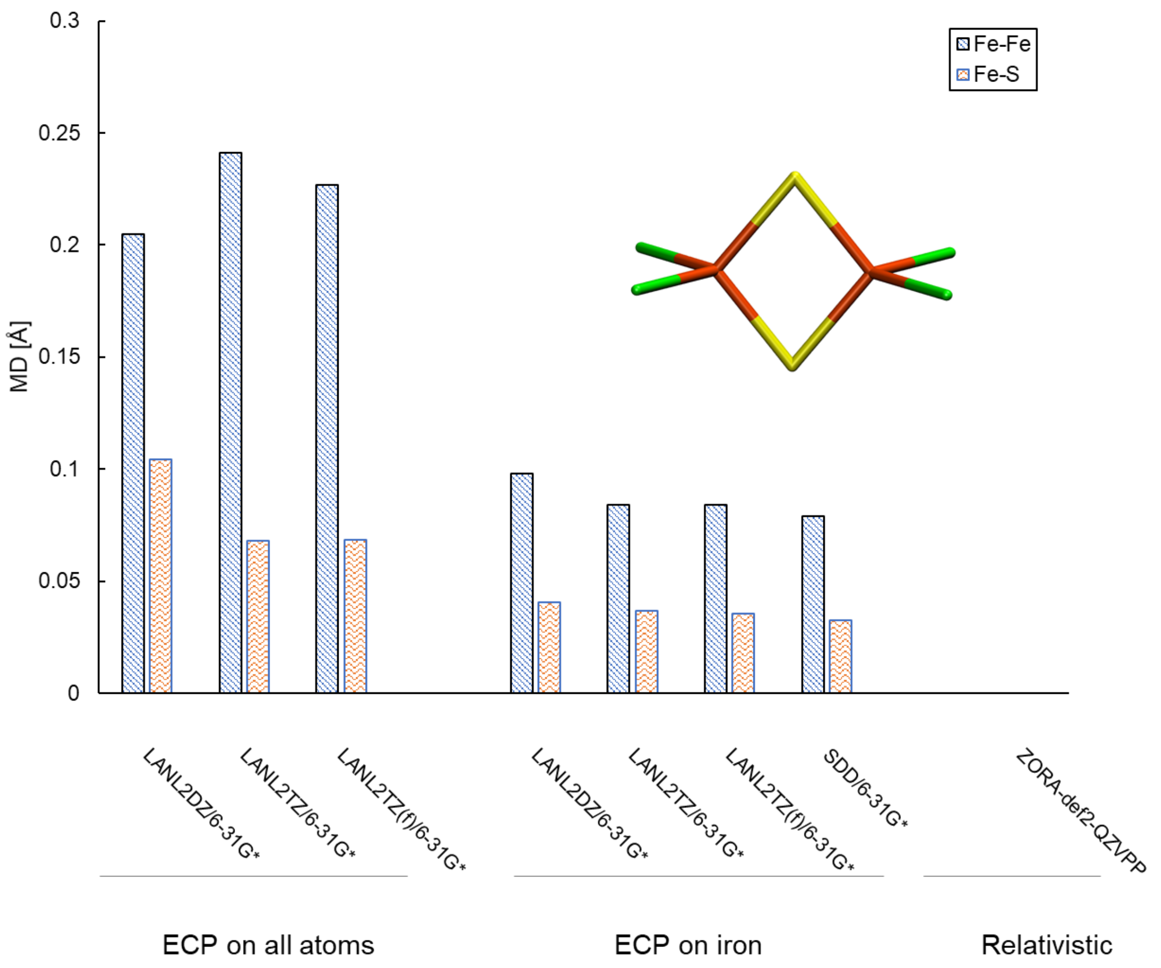

*Figure S9: Mean deviations of Fe-S bond lengths and Fe-Fe distance of complex **7**, $[Fe_2S_2Cl_4]^{2-}$, with or without Hay-Wadt or Stuttgart-Dresden ECPs on all atoms or only on Fe. Deviations are relative to the relativistic ZORA-def2-QZVPP reference (r(Fe-Fe) = 2.690 Å and r_{ave}(Fe-S) = 2.202 Å). The TPSSh functional was used.*

*Table S3: Metal-metal distance and metal-ligand bond lengths (Å) for **1**.*

	Crystal	BP86	PBE	TPSS	B97D3	BLYP	r^{2}SCAN	TPSSh	B3LYP*	B3LYP	PBE0	M06	M06-2X	BHLYP	CAM-B3LYP	ωB97M-D3BJ	ωB97X-D3BJ
Fe1-Fe2	2.702	2.555	2.583	2.578	2.633	2.599	2.677	2.642	2.673	2.714	2.717	2.676	2.843	2.836	2.726	2.734	2.685
Fe1-S1	2.208	2.174	2.180	2.183	2.205	2.199	2.195	2.204	2.218	2.231	2.223	2.242	2.282	2.258	2.224	2.230	2.209
Fe1-S2	2.194	2.161	2.165	2.168	2.186	2.184	2.180	2.188	2.201	2.214	2.208	2.208	2.269	2.248	2.204	2.214	2.193
Fe1-N1	1.990	1.966	1.978	1.974	1.981	1.987	1.965	1.973	1.978	1.980	1.972	1.993	1.994	1.974	1.974	1.980	1.972
Fe1-N2	1.978	1.967	1.978	1.974	1.981	1.987	1.965	1.973	1.978	1.980	1.971	1.961	1.990	1.973	1.974	1.980	1.971
Fe2-S1	2.194	2.161	2.165	2.168	2.186	2.184	2.194	2.188	2.201	2.214	2.208	2.206	2.272	2.248	2.204	2.214	2.193
Fe2-S2	2.208	2.174	2.180	2.183	2.205	2.199	2.212	2.204	2.218	2.231	2.223	2.243	2.279	2.258	2.224	2.232	2.209
Fe2-N3	1.990	1.966	1.978	1.974	1.981	1.987	1.982	1.973	1.978	1.981	1.972	1.961	1.989	1.974	1.974	1.981	1.972
Fe2-N4	1.978	1.967	1.978	1.974	1.982	1.987	1.982	1.973	1.977	1.980	1.971	1.994	1.992	1.973	1.973	1.981	1.971

*Table S4: Metal-metal distance and metal-ligand bond lengths (Å) for **2**.*

	Crystal	BP86	PBE	TPSS	B97D3	BLYP	r^{2}SCAN	TPSSh	B3LYP*	B3LYP	PBE0	M06	M06-2X	BHLYP	CAM-B3LYP	ωB97M-D3BJ	ωB97X-D3BJ
Fe1-Fe2	2.686	2.522	2.573	2.563	2.639	2.591	2.654	2.643	2.690	2.736	2.737	2.674	2.868	2.862	2.755	2.759	2.705
Fe1-S1	2.220	2.201	2.192	2.202	2.234	2.213	2.226	2.253	2.219	2.303	2.302	2.321	2.439	2.372	2.310	2.322	2.294
Fe1-S2	2.232	2.198	2.189	2.198	2.231	2.211	2.224	2.251	2.213	2.304	2.303	2.305	2.406	2.377	2.310	2.325	2.294
Fe1-N1	2.042	1.975	2.031	2.037	2.041	2.048	2.027	2.037	2.013	2.059	2.049	2.024	2.071	2.068	2.054	2.070	2.054
Fe1-N2	2.053	2.053	2.048	2.037	2.053	2.060	2.041	2.045	2.012	2.051	2.041	2.092	2.119	2.059	2.047	2.062	2.047
Fe2-S1	2.232	2.186	2.185	2.189	2.203	2.206	2.203	2.200	2.279	2.219	2.209	2.215	2.248	2.234	2.205	2.212	2.194
Fe2-S2	2.220	2.202	2.193	2.194	2.213	2.214	2.215	2.206	2.273	2.225	2.215	2.228	2.268	2.240	2.213	2.220	2.202
Fe2-N3	2.042	2.022	2.023	2.015	2.028	2.033	2.023	2.015	2.052	2.022	2.011	2.026	2.043	2.009	2.015	2.022	2.012
Fe2-N4	2.053	1.999	2.019	2.015	2.024	2.029	2.019	2.013	2.062	2.025	2.015	2.001	2.022	2.014	2.018	2.025	2.016

*Table S5: Metal-metal distance and metal-ligand bond lengths (Å) for **3**.*

	Crystal	BP86	PBE	TPSS	B97D3	BLYP	r^{2}SCAN	TPSSh	B3LYP*	B3LYP	PBE0	M06	M06-2X	BHLYP	CAM-B3LYP	ωB97M-D3BJ	ωB97X-D3BJ
Fe1-Fe2	2.748	2.611	2.629	2.618	2.703	2.654	2.711	2.701	2.747	2.788	2.791	2.755	2.971	2.913	2.788	2.811	2.756
Fe1-S1	2.267	2.222	2.225	2.237	2.266	2.252	2.248	2.282	2.299	2.320	2.314	2.307	2.405	2.375	2.321	2.333	2.308
Fe1-S2	2.257	2.221	2.222	2.236	2.259	2.246	2.246	2.275	2.292	2.312	2.308	2.306	2.398	2.363	2.313	2.324	2.301
Fe1-N1	2.114	2.079	2.097	2.095	2.113	2.116	2.087	2.105	2.108	2.116	2.104	2.119	2.141	2.122	2.116	2.128	2.108
Fe1-N2	2.115	2.081	2.102	2.095	2.112	2.118	2.093	2.098	2.112	2.121	2.109	2.111	2.146	2.122	2.121	2.132	2.117
Fe2-S1	2.267	2.222	2.225	2.238	2.267	2.253	2.269	2.282	2.299	2.320	2.314	2.307	2.405	2.375	2.321	2.333	2.309
Fe2-S2	2.257	2.221	2.222	2.236	2.259	2.246	2.270	2.275	2.292	2.312	2.308	2.306	2.398	2.363	2.312	2.324	2.301
Fe2-N3	2.114	2.079	2.097	2.095	2.113	2.116	2.121	2.105	2.108	2.116	2.104	2.119	2.140	2.122	2.116	2.129	2.108
Fe2-N4	2.115	2.081	2.102	2.094	2.112	2.118	2.104	2.098	2.112	2.121	2.109	2.111	2.146	2.122	2.121	2.132	2.117

*Table S6: Metal-metal distance and metal-ligand bond lengths (Å) for **4**.*

	Crystal	BP86	PBE	TPSS	B97D3	BLYP	r^{2}SCAN	TPSSh	B3LYP*	B3LYP	PBE0	M06	M06-2X	BHLYP	CAM-B3LYP	ωB97M-D3BJ	ωB97X-D3BJ
Fe1-Fe2	2.679	2.569	2.624	2.591	2.680	2.641	2.728	2.682	2.721	2.763	2.781	2.756	2.910	2.912	2.795	2.795	2.738
Fe1-S1	2.206	2.117	2.186	2.122	2.209	2.204	2.199	2.207	2.220	2.233	2.227	2.229	2.280	2.260	2.227	2.232	2.211
Fe1-S2	2.181	2.108	2.168	2.128	2.188	2.181	2.195	2.196	2.207	2.221	2.224	2.221	2.286	2.260	2.218	2.231	2.202
Fe1-N1	1.996	1.907	2.010	1.920	2.011	2.017	1.991	1.994	2.000	2.003	1.994	1.999	2.008	1.993	1.997	1.999	1.988
Fe1-N2	2.012	1.905	1.997	1.921	2.001	2.006	1.980	1.986	1.993	1.994	1.983	1.991	1.997	1.981	1.984	1.982	1.977
Fe2-S1	2.181	2.110	2.187	2.122	2.212	2.205	2.217	2.208	2.223	2.234	2.227	2.232	2.280	2.259	2.226	2.233	2.212
Fe2-S2	2.206	2.120	2.167	2.128	2.187	2.180	2.207	2.194	2.204	2.222	2.222	2.219	2.281	2.259	2.218	2.229	2.200
Fe2-N3	1.996	1.905	2.010	1.920	2.013	2.019	2.008	1.995	2.002	2.005	1.994	2.001	2.009	1.993	1.997	1.999	1.989
Fe2-N4	2.012	1.909	1.998	1.921	2.000	2.007	1.995	1.985	1.992	1.993	1.981	1.990	1.996	1.980	1.984	1.984	1.976

*Table S7: Metal-metal distance and metal-ligand bond lengths (Å) for **5**.*

	Crystal	BP86	PBE	TPSS	B97D3	BLYP	r^{2}SCAN	TPSSh	B3LYP*	B3LYP	PBE0	M06	M06-2X	BHLYP	CAM-B3LYP	ωB97M-D3BJ	ωB97X-D3BJ
Fe1-Fe2	2.689	2.605	2.635	2.603	2.731	2.641	2.699	2.707	2.773	2.827	2.829	2.811	2.929	2.962	2.865	2.787	2.725
Fe1-S1	2.215	2.238	2.224	2.216	2.303	2.204	2.200	2.300	2.337	2.369	2.352	2.373	2.437	2.435	2.380	2.340	2.303
Fe1-S2	2.233	2.207	2.217	2.221	2.264	2.181	2.182	2.275	2.296	2.324	2.325	2.313	2.413	2.397	2.343	2.329	2.298
Fe1-N1	2.035	1.972	2.017	2.031	1.996	2.017	1.981	2.019	2.024	2.037	2.035	2.027	2.086	2.063	2.044	2.080	2.059
Fe1-N2	2.049	1.977	2.020	2.023	2.005	2.006	1.983	2.022	2.027	2.040	2.037	2.024	2.086	2.062	2.042	2.079	2.058
Fe2-S1	2.227	2.158	2.171	2.174	2.182	2.205	2.197	2.183	2.192	2.201	2.194	2.190	2.252	2.220	2.192	2.209	2.190
Fe2-S2	2.216	2.189	2.192	2.188	2.212	2.180	2.219	2.205	2.217	2.225	2.215	2.228	2.262	2.239	2.211	2.217	2.200
Fe2-N3	2.042	2.002	2.022	2.016	2.027	2.019	1.996	2.010	2.021	2.025	2.018	2.026	2.022	2.015	2.020	2.015	2.005
Fe2-N4	2.045	2.004	2.029	2.018	2.033	2.007	1.998	2.014	2.027	2.031	2.022	2.022	2.028	2.016	2.023	2.017	2.010

*Table S8: Metal-metal distance and metal-ligand bond lengths for **6**.*

	Crystal	BP86	PBE	TPSS	B97D3	BLYP	r^{2}SCAN	TPSSh	B3LYP*	B3LYP	PBE0	M06	M06-2X	BHLYP	CAM-B3LYP	ωB97M-D3BJ	ωB97X-D3BJ
Fe1-Fe2	2.603	2.506	2.534	2.518	2.573	2.546	2.602	2.571	2.601	2.639	2.650	2.652	2.778	2.768	2.657	2.648	2.617
Fe1-C1	2.004	1.952	1.965	1.969	1.984	1.974	1.976	1.985	1.992	2.004	1.999	2.010	2.030	2.025	2.001	2.003	1.987
Fe1-S1	2.199	2.173	2.185	2.189	2.207	2.197	2.203	2.212	2.223	2.236	2.232	2.236	2.305	2.270	2.227	2.234	2.217
Fe1-N1	2.007	1.981	2.005	1.995	2.011	2.010	1.988	1.994	2.001	2.003	1.993	2.002	2.006	1.993	1.996	1.992	1.986
Fe1-N2	1.994	1.976	1.995	1.987	1.998	2.000	1.984	1.989	1.995	1.999	1.991	1.995	2.012	1.990	1.991	1.989	1.984
Fe2-C1	1.984	1.945	1.952	1.951	1.960	1.964	1.972	1.959	1.965	1.974	1.970	1.978	2.017	1.994	1.968	1.968	1.956
Fe2-S1	2.235	2.193	2.208	2.217	2.244	2.220	2.248	2.248	2.265	2.280	2.269	2.272	2.321	2.306	2.271	2.276	2.254
Fe2-N3	2.004	1.982	2.001	1.992	2.003	2.008	2.001	1.991	1.998	2.001	1.990	2.000	2.007	1.994	1.993	1.991	1.985
Fe2-N4	2.003	1.981	2.005	1.996	2.010	2.008	2.010	1.998	2.003	2.007	1.998	2.003	2.016	1.998	1.999	2.001	1.994

*Table S9: Metal-metal distance and metal-ligand bond lengths (Å) for **7.***

	Crystal	BP86	PBE	TPSS	B97D3	BLYP	r^{2}SCAN	TPSSh	B3LYP*	B3LYP	PBE0	M06	M06-2X	BHLYP	CAM-B3LYP	ωB97M-D3BJ	ωB97X-D3BJ
Fe1-Fe2	2.714	2.619	2.624	2.629	2.692	2.656	2.703	2.692	2.725	2.764	2.758	2.734	2.899	2.868	2.771	2.778	2.716
Fe1-Cl1	2.244	2.259	2.260	2.260	2.276	2.286	2.237	2.256	2.267	2.269	2.249	2.250	2.273	2.261	2.255	2.260	2.249
Fe1-Cl3	2.256	2.258	2.260	2.259	2.275	2.285	2.236	2.255	2.266	2.269	2.249	2.250	2.273	2.261	2.255	2.260	2.249
Fe1-S1	2.201	2.180	2.181	2.184	2.207	2.202	2.192	2.203	2.217	2.229	2.220	2.224	2.276	2.252	2.219	2.226	2.203
Fe1-S2	2.198	2.179	2.180	2.183	2.206	2.201	2.191	2.202	2.216	2.227	2.219	2.223	2.274	2.251	2.217	2.224	2.202
Fe2-Cl2	2.244	2.258	2.260	2.259	2.275	2.286	2.262	2.255	2.266	2.269	2.249	2.249	2.273	2.261	2.255	2.263	2.248
Fe2-Cl4	2.256	2.258	2.260	2.259	2.275	2.285	2.261	2.256	2.266	2.269	2.250	2.250	2.273	2.261	2.255	2.263	2.249
Fe2-S1	2.198	2.179	2.180	2.183	2.206	2.201	2.204	2.202	2.216	2.227	2.219	2.223	2.274	2.251	2.217	2.226	2.202
Fe2-N4	2.201	2.180	2.181	2.184	2.207	2.202	2.205	2.203	2.217	2.229	2.220	2.224	2.276	2.252	2.219	2.227	2.203

*Table S10: Metal-metal distance and metal-ligand bond lengths (Å) for **8.***

	Crystal	BP86	PBE	TPSS	B97D3	BLYP	r^{2}SCAN	TPSSh	B3LYP*	B3LYP	PBE0	M06	M06-2X	BHLYP	CAM-B3LYP	ωB97M-D3BJ	ωB97X-D3BJ
Fe1-Fe2	2.695	2.624	2.633	2.631	2.695	2.663	2.710	2.692	2.731	2.769	2.766	2.745	2.931	2.881	2.778	2.784	2.721
Fe1-Ster	2.315	2.296	2.300	2.300	2.319	2.323	2.282	2.304	2.313	2.320	2.304	2.310	2.345	2.327	2.310	2.314	2.297
Fe1-Ster	2.316	2.315	2.320	2.322	2.343	2.348	2.309	2.323	2.336	2.339	2.321	2.336	2.351	2.335	2.323	2.324	2.308
Fe1-Sbr	2.203	2.188	2.189	2.193	2.216	2.211	2.204	2.212	2.227	2.240	2.232	2.243	2.290	2.268	2.230	2.237	2.214
Fe1-Sbr	2.196	2.184	2.187	2.191	2.215	2.208	2.198	2.213	2.228	2.242	2.234	2.235	2.296	2.269	2.233	2.242	2.218
Fe2-Ster	2.316	2.307	2.312	2.313	2.334	2.338	2.327	2.315	2.328	2.333	2.315	2.332	2.345	2.330	2.319	2.325	2.305
Fe2-Ster	2.315	2.309	2.314	2.314	2.332	2.337	2.317	2.314	2.322	2.328	2.310	2.317	2.351	2.327	2.316	2.324	2.303
Fe2-Sbr	2.196	2.179	2.182	2.186	2.209	2.203	2.210	2.209	2.223	2.237	2.230	2.231	2.296	2.266	2.230	2.240	2.214
Fe2-Sbr	2.203	2.177	2.179	2.183	2.206	2.201	2.211	2.205	2.220	2.234	2.227	2.235	2.290	2.264	2.225	2.234	2.209

*Table S11: Metal-metal distance and metal-ligand bond lengths (Å) for **9.***

	Crystal	BP86	PBE	TPSS	B97D3	BLYP	r^{2}SCAN	TPSSh	B3LYP*	B3LYP	PBE0	M06	M06-2X	BHLYP	CAM-B3LYP	ωB97M-D3BJ	ωB97X-D3BJ
Fe1-Fe2	2.508	2.495	2.508	2.493	2.513	2.544	2.493	2.485	2.498	2.501	2.482	2.504	2.697	2.655	2.474	2.579	2.489
Fe1-O1	1.983	2.042	2.046	2.035	2.043	2.072	2.030	2.027	2.034	2.033	2.022	2.020	1.969	2.139	2.020	2.125	2.065
Fe1-O2	1.915	2.042	2.045	2.034	2.044	2.073	2.029	2.027	2.035	2.036	2.024	2.022	1.970	2.144	2.022	2.131	2.070
Fe1-O3	1.933	2.041	2.044	2.034	2.041	2.070	2.029	2.025	2.033	2.032	2.019	2.018	1.970	2.140	2.018	2.127	2.066
Fe1-N1	2.195	2.220	2.232	2.212	2.240	2.258	2.214	2.208	2.225	2.229	2.215	2.219	2.215	2.206	2.220	2.210	2.202
Fe1-N2	2.224	2.221	2.232	2.212	2.241	2.258	2.215	2.208	2.223	2.227	2.211	2.219	2.214	2.204	2.217	2.211	2.199
Fe1-N3	2.216	2.221	2.233	2.214	2.242	2.260	2.216	2.209	2.225	2.228	2.214	2.219	2.216	2.205	2.218	2.211	2.202
Fe2-O1	1.933	2.039	2.042	2.032	2.042	2.072	2.025	2.022	2.031	2.030	2.014	2.017	2.156	1.949	2.008	1.969	1.986
Fe2-O2	1.909	2.043	2.046	2.035	2.044	2.074	2.028	2.025	2.034	2.033	2.017	2.020	2.159	1.950	2.012	1.970	1.989
Fe2-O3	1.983	2.038	2.042	2.032	2.040	2.068	2.026	2.022	2.030	2.029	2.014	2.017	2.152	1.948	2.008	1.969	1.989
Fe2-N4	2.195	2.218	2.231	2.211	2.238	2.260	2.214	2.206	2.222	2.225	2.211	2.220	2.227	2.200	2.216	2.214	2.206
Fe2-N5	2.224	2.218	2.230	2.211	2.238	2.260	2.214	2.207	2.222	2.226	2.211	2.220	2.229	2.202	2.217	2.213	2.207
Fe2-N6	2.216	2.218	2.229	2.209	2.237	2.258	2.212	2.205	2.221	2.224	2.209	2.217	2.227	2.201	2.215	2.211	2.205

Table S12: Metal-metal distance and metal-ligand bond lengths (Å) for ***10.***

	Crystal	BP86	PBE	TPSS	B97D3	BLYP	r²SCAN	TPSSh	B3LYP*	B3LYP	PBE0	M06	M06-2X	BHLYP	CAM-B3LYP	ωB97M-D3BJ	ωB97X-D3BJ
Mo1-Fe1	2.765	2.721	2.726	2.711	2.775	2.763	2.786	2.734	2.779	2.802	2.780	2.802	2.966	3.006	2.807	2.792	2.713
Mo1-O1	1.677	1.707	1.705	1.706	1.699	1.719	1.693	1.693	1.696	1.692	1.675	1.676	1.662	1.661	1.674	1.667	1.665
Mo1-S1	2.292	2.315	2.315	2.315	2.317	2.335	2.309	2.305	2.315	2.316	2.301	2.323	2.358	2.370	2.312	2.310	2.283
Mo1-S2	2.312	2.318	2.316	2.317	2.319	2.337	2.303	2.304	2.308	2.308	2.294	2.318	2.358	2.361	2.303	2.303	2.285
Mo1-O2	2.044	2.068	2.070	2.062	2.074	2.087	2.061	2.055	2.070	2.068	2.048	2.053	2.042	2.028	2.048	2.056	2.047
Mo1-O3	2.073	2.073	2.074	2.067	2.077	2.090	2.055	2.055	2.061	2.058	2.039	2.052	2.041	2.020	2.039	2.048	2.051
Fe1-Cl1	2.250	2.257	2.259	2.261	2.274	2.282	2.255	2.259	2.266	2.271	2.255	2.256	2.281	2.261	2.260	2.273	2.259
Fe1-Cl2	2.236	2.246	2.248	2.250	2.267	2.273	2.258	2.252	2.260	2.265	2.247	2.241	2.256	2.251	2.252	2.261	2.252
Fe1-S1	2.225	2.209	2.210	2.213	2.229	2.233	2.222	2.226	2.238	2.247	2.232	2.239	2.272	2.239	2.230	2.240	2.217
Fe1-S2	2.224	2.208	2.209	2.211	2.228	2.232	2.225	2.226	2.243	2.253	2.237	2.240	2.271	2.247	2.237	2.246	2.214

Table S13: Metal-metal distance and metal-ligand bond lengths (Å) for ***11.***

	Crystal	BP86	PBE	TPSS	B97D3	BLYP	r²SCAN	TPSSh	B3LYP*	B3LYP	PBE0	M06	M06-2X	BHLYP	CAM-B3LYP	ωB97M-D3BJ	ωB97X-D3BJ
Fe1-Mo1	2.756	2.699	2.710	2.694	2.741	2.740	2.743	2.718	2.758	2.782	2.760	2.784	2.982	2.915	2.802	2.815	2.710
Fe1-S3	2.261	2.237	2.238	2.244	2.266	2.267	2.258	2.270	2.290	2.312	2.297	2.296	2.449	2.414	2.322	2.347	2.286
Fe1-S4	2.267	2.233	2.234	2.236	2.256	2.256	2.257	2.258	2.279	2.301	2.288	2.295	2.452	2.406	2.318	2.339	2.277
Fe1-S5	2.299	2.268	2.274	2.278	2.296	2.296	2.297	2.294	2.303	2.315	2.308	2.317	2.387	2.352	2.315	2.330	2.302
Fe1-S6	2.316	2.277	2.287	2.288	2.306	2.307	2.305	2.298	2.308	2.319	2.312	2.314	2.393	2.353	2.313	2.331	2.306
Mo1-S1	2.148	2.180	2.181	2.179	2.177	2.197	2.168	2.167	2.172	2.169	2.151	2.162	2.145	2.139	2.147	2.142	2.134
Mo1-S2	2.156	2.182	2.178	2.176	2.176	2.195	2.170	2.165	2.171	2.168	2.150	2.166	2.141	2.144	2.150	2.141	2.133
Mo1-S3	2.252	2.271	2.274	2.273	2.271	2.293	2.261	2.260	2.265	2.260	2.241	2.259	2.225	2.221	2.237	2.236	2.224
Mo1-S4	2.255	2.266	2.271	2.269	2.268	2.289	2.260	2.255	2.261	2.257	2.238	2.261	2.226	2.217	2.234	2.234	2.220

Table S14: Metal-metal distance and metal-ligand bond lengths for (Å) ***D1.***

	Crystal	BP86	PBE	TPSS	B97D3	BLYP	r²SCAN	TPSSh	B3LYP*	B3LYP	PBE0	M06	M06-2X	BHLYP	CAM-B3LYP	ωB97M-D3BJ	ωB97X-D3BJ
Fe1-Fe2	2.589	2.581	2.589	2.579	2.605	2.628	2.594	2.571	2.601	2.607	2.569	2.592	2.625	2.606	2.589	2.577	2.554
Fe1-H1	1.670	1.663	1.668	1.660	1.658	1.670	1.656	1.652	1.653	1.652	1.646	1.657	1.676	1.651	1.641	1.648	1.643
Fe1-P1	2.272	2.236	2.251	2.246	2.257	2.279	2.254	2.244	2.263	2.274	2.245	2.281	2.375	2.345	2.281	2.262	2.230
Fe1-C17	1.781	1.753	1.753	1.764	1.751	1.770	1.749	1.767	1.770	1.779	1.764	1.783	1.888	1.859	1.787	1.801	1.784
Fe1-C18	1.784	1.756	1.755	1.766	1.754	1.774	1.752	1.770	1.773	1.784	1.767	1.785	1.891	1.862	1.790	1.806	1.789
Fe1-S1	2.278	2.289	2.288	2.287	2.307	2.328	2.285	2.283	2.308	2.315	2.278	2.296	2.389	2.344	2.298	2.301	2.270
Fe1-S2	2.268	2.283	2.282	2.281	2.298	2.318	2.286	2.276	2.299	2.306	2.272	2.300	2.386	2.344	2.298	2.295	2.265
Fe2-H1	1.763	1.663	1.668	1.660	1.657	1.670	1.655	1.651	1.653	1.651	1.645	1.657	1.673	1.649	1.640	1.648	1.643
Fe2-S1	2.286	2.290	2.289	2.288	2.309	2.330	2.287	2.284	2.309	2.316	2.279	2.296	2.395	2.346	2.300	2.302	2.270
Fe2-S2	2.283	2.281	2.281	2.280	2.297	2.317	2.285	2.275	2.298	2.304	2.271	2.301	2.382	2.344	2.297	2.293	2.264
Fe2-P2	2.259	2.236	2.250	2.245	2.257	2.278	2.254	2.243	2.263	2.274	2.244	2.279	2.368	2.340	2.279	2.262	2.230
Fe2-C39	1.791	1.753	1.753	1.763	1.750	1.770	1.749	1.767	1.769	1.779	1.764	1.782	1.885	1.857	1.786	1.801	1.784
Fe2-C40	1.793	1.756	1.755	1.766	1.754	1.773	1.752	1.770	1.773	1.783	1.767	1.785	1.892	1.863	1.791	1.805	1.789

*Table S15: Metal-metal distance and metal-ligand bond lengths (Å) for **D2.***

	Crystal	BP86	PBE	TPSS	B97D3	BLYP	r^{2}SCAN	TPSSh	B3LYP*	B3LYP	PBE0	M06	M06-2X	BHLYP	CAM-B3LYP	ωB97M-D3BJ	ωB97X-D3BJ
Fe1-Fe1	3.192	3.099	3.137	3.106	3.150	3.156	3.165	3.094	3.125	3.131	3.100	3.119	3.104	3.119	3.131	3.104	3.069
Fe1-S1	2.342	2.316	2.314	2.319	2.344	2.360	2.322	2.316	2.344	2.353	2.312	2.327	2.439	2.387	2.342	2.345	2.304
Fe1-S2	2.332	2.329	2.334	2.334	2.363	2.376	2.352	2.333	2.361	2.374	2.335	2.369	2.442	2.414	2.367	2.369	2.329
Fe1-S3	2.325	2.292	2.306	2.302	2.320	2.339	2.323	2.301	2.327	2.338	2.304	2.340	2.413	2.374	2.337	2.336	2.297
Fe1-P4	2.253	2.190	2.214	2.203	2.210	2.232	2.231	2.212	2.238	2.260	2.237	2.275	2.402	2.377	2.283	2.274	2.225
Fe1-P5	2.230	2.147	2.161	2.155	2.156	2.180	2.168	2.159	2.179	2.193	2.173	2.196	2.394	2.272	2.200	2.199	2.161
Fe1-P6	2.236	2.171	2.186	2.178	2.184	2.208	2.196	2.185	2.211	2.228	2.204	2.238	2.307	2.325	2.241	2.236	2.194
Fe2-S1	2.362	2.319	2.324	2.325	2.351	2.364	2.337	2.324	2.350	2.361	2.323	2.342	2.450	2.400	2.353	2.358	2.317
Fe2-S2	2.345	2.327	2.328	2.330	2.357	2.373	2.340	2.327	2.356	2.367	2.326	2.358	2.436	2.399	2.356	2.355	2.316
Fe2-S3	2.335	2.294	2.310	2.305	2.323	2.340	2.333	2.305	2.330	2.342	2.311	2.354	2.427	2.401	2.345	2.348	2.306
Fe2-P1	2.231	2.178	2.194	2.186	2.192	2.215	2.209	2.193	2.219	2.237	2.214	2.269	2.379	2.347	2.254	2.247	2.204
Fe2-P2	2.204	2.150	2.163	2.158	2.159	2.184	2.170	2.162	2.182	2.196	2.175	2.203	2.299	2.274	2.203	2.201	2.163
Fe2-P3	2.235	2.194	2.213	2.205	2.211	2.234	2.229	2.214	2.241	2.263	2.239	2.276	2.433	2.386	2.286	2.278	2.229

*Table S16: Metal-metal distance and metal-ligand bond lengths (Å) for **D3.***

	Crystal	BP86	PBE	TPSS	B97D3	BLYP	r^{2}SCAN	TPSSh	B3LYP*	B3LYP	PBE0	M06	M06-2X	BHLYP	CAM-B3LYP	ωB97M-D3BJ	ωB97X-D3BJ
Fe1-Fe2	2.523	2.497	2.494	2.495	2.513	2.541	2.482	2.480	2.504	2.505	2.459	2.485	2.505	2.482	2.474	2.463	2.443
Fe1-C1	2.013	1.995	1.993	1.996	2.004	2.022	1.983	1.987	1.997	1.999	1.971	1.987	1.994	1.987	1.975	1.969	1.960
Fe1-C2	1.836	1.806	1.804	1.812	1.812	1.827	1.796	1.808	1.813	1.816	1.795	1.817	1.829	1.826	1.812	1.817	1.810
Fe1-C3	2.013	1.995	1.993	1.996	2.004	2.022	1.983	1.987	1.997	1.998	1.971	1.986	1.994	1.986	1.975	1.969	1.960
Fe1-C4	2.013	1.995	1.994	1.996	2.003	2.022	1.984	1.986	1.997	1.998	1.970	1.985	1.993	1.985	1.975	1.969	1.960
Fe1-C5	1.836	1.806	1.804	1.812	1.812	1.827	1.796	1.808	1.813	1.816	1.794	1.816	1.828	1.826	1.812	1.817	1.810
Fe1-C6	1.835	1.805	1.803	1.812	1.811	1.826	1.795	1.807	1.812	1.815	1.793	1.815	1.826	1.823	1.811	1.815	1.808
Fe2-C1	2.013	1.995	1.994	1.996	2.004	2.022	1.983	1.987	1.997	1.999	1.971	1.987	1.994	1.986	1.975	1.969	1.960
Fe2-C3	2.013	1.995	1.993	1.996	2.004	2.022	1.984	1.987	1.997	1.998	1.971	1.986	1.994	1.986	1.975	1.969	1.960
Fe2-C4	2.013	1.995	1.994	1.996	2.003	2.022	1.983	1.986	1.997	1.998	1.970	1.985	1.993	1.984	1.975	1.969	1.960
Fe2-C7	1.836	1.806	1.804	1.812	1.812	1.827	1.796	1.808	1.813	1.816	1.795	1.817	1.829	1.826	1.812	1.817	1.810
Fe2-C8	1.836	1.806	1.804	1.812	1.812	1.827	1.796	1.808	1.813	1.816	1.794	1.816	1.828	1.826	1.812	1.817	1.810
Fe2-C9	1.835	1.805	1.803	1.812	1.811	1.826	1.795	1.807	1.812	1.815	1.793	1.815	1.826	1.823	1.811	1.815	1.808

*Table S17: Metal-metal distance and metal-ligand bond lengths (Å) for **D4.***

	Crystal	BP86	PBE	TPSS	B97D3	BLYP	r^2SCAN	TPSSh	B3LYP*	B3LYP	PBE0	M06	M06-2X	BHLYP	CAM-B3LYP	ωB97M-D3BJ	ωB97X-D3BJ
Fe1-Fe2	2.509	2.525	2.530	2.506	2.538	2.556	2.506	2.484	2.507	2.501	2.473	2.482	2.486	2.465	2.469	2.450	2.431
Fe1-C1	1.945	1.889	1.889	1.900	1.901	1.918	1.884	1.897	1.905	1.912	1.885	1.916	1.973	1.938	1.914	1.922	1.912
Fe1-C2	1.755	1.736	1.736	1.743	1.733	1.752	1.728	1.742	1.745	1.751	1.735	1.751	1.812	1.798	1.748	1.760	1.747
Fe1-C3	1.741	1.736	1.735	1.743	1.733	1.752	1.728	1.741	1.745	1.750	1.735	1.751	1.813	1.798	1.748	1.759	1.746
Fe1-S1	2.288	2.288	2.287	2.286	2.307	2.327	2.288	2.283	2.308	2.317	2.279	2.304	2.403	2.357	2.301	2.305	2.275
Fe1-S2	2.278	2.288	2.287	2.286	2.307	2.327	2.287	2.282	2.308	2.317	2.279	2.303	2.403	2.356	2.302	2.305	2.274
Fe2-C4	1.743	1.739	1.738	1.745	1.736	1.755	1.731	1.744	1.747	1.753	1.738	1.753	1.818	1.800	1.750	1.762	1.748
Fe2-C5	1.939	1.886	1.887	1.897	1.898	1.915	1.881	1.895	1.903	1.910	1.883	1.913	1.969	1.936	1.911	1.919	1.910
Fe2-C6	1.745	1.739	1.738	1.745	1.736	1.754	1.731	1.744	1.747	1.752	1.737	1.753	1.818	1.800	1.750	1.761	1.748
Fe2-S1	2.290	2.282	2.282	2.281	2.301	2.321	2.280	2.276	2.301	2.308	2.271	2.296	2.388	2.343	2.292	2.294	2.265
Fe2-S2	2.277	2.282	2.281	2.281	2.301	2.321	2.281	2.276	2.300	2.308	2.270	2.296	2.390	2.346	2.292	2.294	2.265

*Table S18: Metal-metal distance and metal-ligand bond lengths (Å) for **D5.***

	Crystal	BP86	PBE	TPSS	B97D3	BLYP	r^2SCAN	TPSSh	B3LYP*	B3LYP	PBE0	M06	M06-2X	BHLYP	CAM-B3LYP	ωB97M-D3BJ	ωB97X-D3BJ
Fe-Fe	2.505	2.510	2.476	2.492	2.525	2.476	2.491	2.476	2.502	2.499	2.468	2.468	2.472	2.459	2.468	2.450	2.432
Fe1-CO	1.804	1.768	1.773	1.776	1.772	1.773	1.760	1.773	1.778	1.783	1.762	1.785	1.829	1.814	1.782	1.790	1.780
Fe1-CO	1.793	1.771	1.779	1.780	1.773	1.779	1.764	1.779	1.783	1.789	1.772	1.787	1.844	1.827	1.788	1.797	1.786
Fe1-CO	1.802	1.772	1.779	1.780	1.773	1.779	1.764	1.779	1.783	1.789	1.771	1.788	1.842	1.825	1.787	1.796	1.785
Fe1-S	2.266	2.275	2.265	2.272	2.289	2.265	2.271	2.265	2.287	2.292	2.255	2.283	2.357	2.313	2.272	2.273	2.246
Fe1-S	2.266	2.276	2.265	2.273	2.289	2.265	2.270	2.265	2.286	2.290	2.255	2.281	2.358	2.310	2.269	2.271	2.245
Fe2-CO	1.824	1.774	1.780	1.782	1.778	1.780	1.767	1.780	1.785	1.790	1.769	1.793	1.849	1.826	1.789	1.800	1.789
Fe2-CO	1.797	1.767	1.776	1.776	1.769	1.776	1.761	1.776	1.780	1.786	1.768	1.783	1.840	1.827	1.785	1.795	1.783
Fe2-S	2.255	2.269	2.260	2.266	2.281	2.260	2.266	2.260	2.282	2.287	2.253	2.283	2.376	2.316	2.270	2.272	2.245
Fe2-S	2.277	2.284	2.274	2.280	2.299	2.274	2.282	2.274	2.297	2.303	2.267	2.294	2.381	2.330	2.284	2.289	2.260
Fe2-CO	1.789	1.765	1.772	1.773	1.765	1.772	1.757	1.772	1.776	1.782	1.764	1.780	1.834	1.820	1.781	1.789	1.777

Table S19: Metal-metal distance and metal-ligand bond lengths (Å) for the 244 QM atom QM/MM model of FeMoco.

	X-ray	X-ray	Ave X Ray	BP86	PBE	TPSS	B97D3	BLYP	r^2SCAN	TPSSh	B3LYP*	B3LYP	PBE0	M06	M06-2X	BHLYP	CAM-B3LYP	ωB97M-D3BJ	ωB97X-D3BJ
Mo_C	3.530	3.550	3.540	3.466	3.476	3.459	3.507	3.553	3.516	3.496	3.545	3.594	3.587	3.614	3.872	3.854	3.631	3.659	3.562
C_Fe1	3.467	3.448	3.458	3.373	3.385	3.365	3.434	3.462	3.454	3.451	3.496	3.539	3.534	3.529	3.764	3.748	3.594	3.603	3.529
Mo_Fe1	6.996	6.997	6.997	6.836	6.858	6.822	6.938	7.012	6.969	6.946	7.040	7.132	7.121	7.141	7.635	7.600	7.224	7.261	7.089
Fe1_Fe2	2.668	2.669	2.668	2.554	2.558	2.551	2.626	2.615	2.649	2.655	2.708	2.756	2.747	2.749	2.981	2.977	2.802	2.803	2.737
Fe1_Fe3	2.663	2.668	2.665	2.553	2.561	2.548	2.623	2.633	2.644	2.633	2.683	2.723	2.711	2.698	2.885	2.877	2.766	2.762	2.698
Fe1_Fe4	2.662	2.655	2.658	2.650	2.654	2.622	2.672	2.721	2.663	2.643	2.681	2.701	2.689	2.685	2.903	2.908	2.758	2.764	2.694
Fe2_Fe3	2.667	2.672	2.669	2.575	2.573	2.580	2.665	2.639	2.659	2.659	2.717	2.757	2.726	2.745	2.985	2.986	2.770	2.805	2.702
Fe2_Fe4	2.654	2.654	2.654	2.565	2.567	2.561	2.622	2.620	2.633	2.635	2.682	2.715	2.705	2.720	2.869	2.834	2.729	2.732	2.679
Fe3_Fe4	2.644	2.641	2.642	2.552	2.556	2.550	2.605	2.609	2.614	2.612	2.651	2.686	2.679	2.681	2.913	2.851	2.717	2.735	2.667
Fe2_Fe6	2.580	2.576	2.578	2.517	2.524	2.527	2.562	2.568	2.580	2.587	2.599	2.609	2.609	2.630	2.756	2.737	2.597	2.625	2.585
Fe4_Fe5	2.612	2.612	2.612	2.551	2.550	2.563	2.583	2.599	2.597	2.594	2.606	2.633	2.645	2.649	2.757	2.690	2.645	2.668	2.636
Fe3_Fe7	2.589	2.583	2.586	2.537	2.563	2.551	2.584	2.600	2.597	2.597	2.614	2.633	2.634	2.636	2.746	2.688	2.629	2.650	2.619
Fe5_Fe6	2.630	2.632	2.631	2.548	2.553	2.544	2.607	2.616	2.619	2.627	2.678	2.712	2.700	2.710	2.993	2.915	2.724	2.736	2.665
Fe5_Fe7	2.631	2.627	2.629	2.564	2.563	2.551	2.606	2.614	2.609	2.616	2.660	2.688	2.676	2.695	2.855	2.755	2.691	2.693	2.645
Fe6_Fe7	2.595	2.601	2.598	2.522	2.523	2.513	2.575	2.574	2.574	2.568	2.618	2.652	2.629	2.658	2.896	2.907	2.673	2.691	2.604
Mo_Fe6	2.667	2.674	2.671	2.618	2.620	2.605	2.640	2.672	2.651	2.646	2.696	2.755	2.753	2.769	3.022	3.036	2.814	2.835	2.735
Mo_Fe7	2.676	2.681	2.679	2.606	2.615	2.592	2.634	2.680	2.635	2.620	2.671	2.724	2.713	2.727	3.080	3.054	2.781	2.813	2.697
Mo_Fe5	2.725	2.727	2.726	2.677	2.679	2.664	2.725	2.753	2.722	2.710	2.761	2.786	2.764	2.807	2.952	2.927	2.788	2.797	2.728
Fe1_S1	2.306	2.295	2.300	2.259	2.260	2.272	2.300	2.305	2.300	2.308	2.325	2.347	2.343	2.336	2.483	2.472	2.394	2.414	2.367
Fe1_S2	2.270	2.266	2.268	2.188	2.190	2.201	2.222	2.222	2.234	2.237	2.252	2.271	2.274	2.283	2.386	2.353	2.283	2.295	2.265
Fe1_S3	2.289	2.287	2.288	2.256	2.258	2.265	2.298	2.309	2.298	2.301	2.324	2.341	2.329	2.328	2.482	2.435	2.368	2.385	2.340
Fe2_S1	2.263	2.261	2.262	2.164	2.169	2.180	2.215	2.208	2.227	2.247	2.268	2.295	2.294	2.302	2.381	2.348	2.295	2.309	2.283
Fe2_S2	2.246	2.254	2.250	2.186	2.192	2.202	2.239	2.245	2.246	2.258	2.275	2.283	2.271	2.294	2.318	2.300	2.263	2.276	2.254
Fe2_S7	2.198	2.206	2.202	2.152	2.157	2.159	2.180	2.184	2.181	2.192	2.207	2.220	2.214	2.234	2.265	2.231	2.206	2.217	2.200
Fe3_S2	2.267	2.275	2.271	2.226	2.230	2.243	2.288	2.280	2.292	2.301	2.328	2.356	2.343	2.342	2.480	2.423	2.375	2.405	2.346
Fe3_S3	2.251	2.254	2.253	2.175	2.181	2.191	2.221	2.227	2.231	2.247	2.266	2.291	2.296	2.300	2.397	2.360	2.305	2.313	2.288
Fe3_S9	2.229	2.221	2.225	2.183	2.189	2.192	2.211	2.227	2.212	2.221	2.233	2.250	2.251	2.253	2.342	2.302	2.251	2.271	2.248
Fe4_S1	2.286	2.286	2.286	2.257	2.264	2.266	2.296	2.314	2.298	2.285	2.300	2.300	2.286	2.318	2.321	2.300	2.270	2.277	2.255
Fe4_S3	2.286	2.287	2.286	2.261	2.271	2.273	2.305	2.320	2.303	2.295	2.311	2.316	2.301	2.318	2.336	2.311	2.289	2.302	2.275
Fe4_S8	2.240	2.240	2.240	2.196	2.209	2.215	2.240	2.251	2.248	2.244	2.258	2.271	2.266	2.280	2.303	2.270	2.255	2.260	2.246
Fe5_S4	2.261	2.268	2.264	2.216	2.220	2.224	2.257	2.263	2.261	2.265	2.292	2.311	2.297	2.312	2.453	2.422	2.322	2.350	2.291
Fe5_S6	2.256	2.250	2.253	2.207	2.216	2.221	2.253	2.263	2.253	2.264	2.291	2.310	2.297	2.309	2.468	2.425	2.311	2.339	2.290
Fe5_S8	2.261	2.261	2.261	2.192	2.204	2.206	2.225	2.242	2.221	2.226	2.239	2.252	2.248	2.248	2.344	2.311	2.249	2.268	2.242
Fe6_S4	2.237	2.234	2.236	2.189	2.192	2.193	2.216	2.234	2.232	2.234	2.257	2.284	2.284	2.293	2.398	2.366	2.294	2.313	2.269
Fe6_S5	2.220	2.219	2.220	2.175	2.179	2.187	2.209	2.224	2.222	2.226	2.242	2.260	2.252	2.260	2.383	2.353	2.267	2.292	2.244
Fe6_S7	2.178	2.173	2.175	2.147	2.153	2.157	2.171	2.188	2.184	2.186	2.194	2.206	2.208	2.212	2.287	2.254	2.206	2.220	2.200
Fe7_S5	2.253	2.246	2.250	2.231	2.232	2.239	2.265	2.272	2.271	2.278	2.304	2.320	2.309	2.322	2.377	2.338	2.308	2.323	2.296
Fe7_S6	2.221	2.220	2.220	2.180	2.183	2.185	2.208	2.218	2.213	2.210	2.233	2.255	2.248	2.259	2.404	2.359	2.266	2.287	2.240
Fe7_S9	2.213	2.210	2.212	2.167	2.174	2.175	2.195	2.208	2.202	2.200	2.214	2.224	2.218	2.230	2.255	2.233	2.214	2.222	2.213
Mo_S4	2.358	2.348	2.353	2.329	2.329	2.333	2.328	2.358	2.327	2.328	2.334	2.333	2.318	2.352	2.331	2.319	2.316	2.314	2.302
Mo_S5	2.371	2.367	2.369	2.367	2.362	2.369	2.367	2.391	2.366	2.368	2.381	2.397	2.390	2.406	2.482	2.475	2.420	2.425	2.380
Mo_S6	2.354	2.354	2.354	2.330	2.333	2.336	2.335	2.364	2.330	2.327	2.337	2.340	2.325	2.356	2.318	2.307	2.324	2.319	2.307
C_Fe2	2.014	1.997	2.005	1.926	1.931	1.931	1.979	1.974	1.983	1.991	2.019	2.034	2.025	2.050	2.069	2.056	2.024	2.023	1.996
C_Fe3	1.990	1.984	1.987	1.943	1.945	1.945	1.991	1.984	1.995	2.010	2.042	2.083	2.074	2.074	2.370	2.324	2.115	2.162	2.072
C_Fe4	2.003	1.991	1.997	1.952	1.958	1.957	1.980	1.992	1.992	1.983	2.001	2.016	2.018	2.019	2.092	2.060	2.115	2.036	2.009
C_Fe5	2.006	2.008	2.007	1.960	1.963	1.958	1.980	1.999	1.969	1.972	1.990	2.001	2.001	2.004	2.153	2.064	2.004	2.012	1.986
C_Fe6	2.008	2.018	2.013	1.941	1.944	1.944	1.988	1.986	2.009	2.004	2.035	2.058	2.047	2.060	2.328	2.331	2.065	2.093	2.023
C_Fe7	1.979	1.997	1.988	1.946	1.951	1.946	1.986	1.990	1.998	1.992	2.019	2.040	2.034	2.051	2.022	1.991	2.039	2.028	2.015
